# 20.4% Power conversion efficiency from albedo-collecting organic solar cells under 0.2 albedo

**DOI:** 10.1126/sciadv.adp9439

**Published:** 2024-11-01

**Authors:** Hao Ren, Jing-De Chen, Ye-Fan Zhang, Jia-Liang Zhang, Wei-Shuo Chen, Yan-Qing Li, Jian-Xin Tang

**Affiliations:** ^1^ Macao Institute of Materials Science and Engineering (MIMSE), Faculty of Innovation Engineering (FIE), Macau University of Science and Technology, Taipa, Macao SAR 999078, China.; ^2^ Jiangsu Key Laboratory for Carbon-Based Functional Materials and Devices, Institute of Functional Nano and Soft Materials (FUNSOM), Soochow University, Suzhou, Jiangsu 215123, China.; ^3^ School of Physics and Electronic Science, East China Normal University, Shanghai 200241, China.

## Abstract

Highly efficient bifacial organic solar cells (OSCs) have not been reported due to limited thickness of the active layer in conventional configurations, not allowing for efficient harvesting of front sunlight and albedo light. Here, bifacial OSCs are reported with efficiency higher than the monofacial counterparts. The incorporation of pyramid-based asymmetrical optical transmission (AOT) array to a transparent silver electrode suppresses the escaping of front sunlight without sacrificing the harvesting of albedo light. Parasitic absorption induced by the excitation of surface plasmons in an AOT electrode is further reduced by doping organic emitter in electron transport layer and capping high dielectric constant film to silver. The rear electrode achieves a front transmittance of 7% and a rear transmission of 86%. At a conventional albedo of 0.2, the synergistic effect of AOT and minimized optical loss endow the bifacial OSCs with power conversion efficiency of 20.4%. This work paves the way for the utilization of albedo light in OSCs.

## Teaser

The incorporation of pyramid-based asymmetrical optical transmission array boosts the efficiency of bifacial organic solar cells.

## INTRODUCTION

Organic solar cells (OSCs) are perceived as one of the most promising next-generation sustainable energy technologies due to their unique features like light weight, flexibility, transparency, low cost, and easy processing (*1*–*3*). To date, the power conversion efficiencies (PCEs) of the rigid and flexible single-junction OSCs exceed 20 and 18%, respectively (*4*–*9*). The strides made in high-efficiency OSCs were predicated on the development of organic semiconductors and device structures that improve the efficiency of sunlight utilization. The employment of narrow bandgap nonfullerene acceptor materials, such as ITIC, Y-series acceptors, M36, etc., has greatly boosted the absorption edge from visible to near-infrared region (*10*–*16*). Combined with high-performance donor materials and multicomponent systems, light harvesting in a broad spectrum was implemented, producing continuous efficiency breakthroughs.

In addition to a broad absorption spectrum, efficient light harvesting in the active layer is another vital issue to achieving high light utilization efficiency (LUE). However, substantial optical losses composed of reflection loss at the interfaces, parasitic absorption in functional layers, and insufficient light absorption in the active layer limit the performance of OSCs (*17*–*21*). Accordingly, optical manipulation strategies have been well exploited to mitigate the optical losses in OSCs. The optical field distribution in active layer can be enhanced by virtue of the presence of optical phenomena such as antireflection, surface plasmon (SP) resonance, waveguide mode, or scattering, thereby improving light harvesting (*22*–*25*). Metal nanoparticle (*26*, *27*), subwavelength grating (*28*), blazed grating (*16*), moth-eye structure (*29*, *30*), microcavity (*31*), etc. have been explored to realize a broadband and efficient light harvesting of OSCs. The peak absorption of OSCs can exceed 95% through rational optical design of the device structure (*32*). However, optical design of OSCs is now facing a bottleneck because it is difficult to further improve the absorption of front sunlight.

**Fig. 1.**
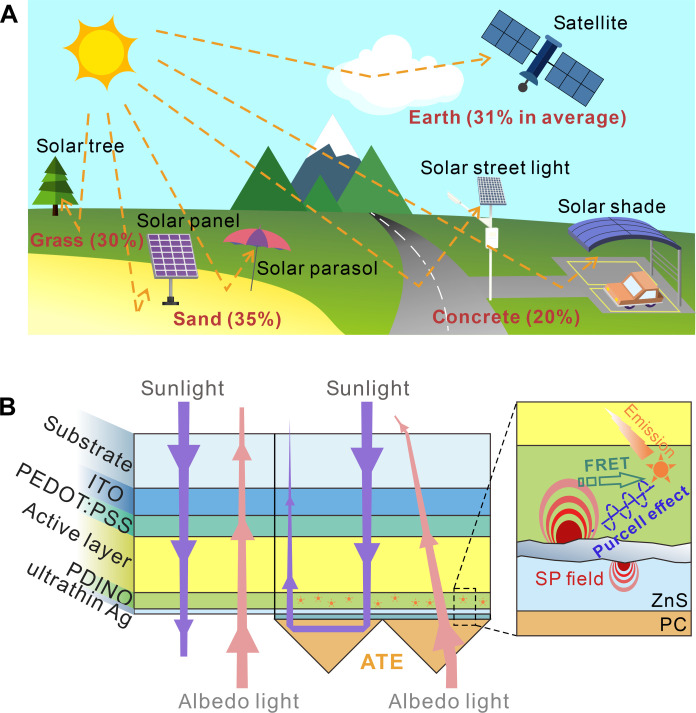
The advantages of bifacial OSCs and ATE. (**A**) Schematic view of albedo light and representative applications of bifacial OSCs. The percentages represent the albedo values of surrounding surfaces. (**B**) Schematic device structures and ray tracing diagrams of bifacial OSCs with the ultrathin Ag electrode (left) and ATE (right). Enlarged is a schematic of SP-enhanced emission and SP regulation. FRET, Förster resonance energy transfer.

It is worth noting that the most efficient OSCs are based on monofacial light-harvesting structure with an opaque rear electrode, through which the escaping of unabsorbed sunlight can be suppressed. However, it has been realized that surfaces in surrounding environments reflect and diffuse a large portion of sunlight, namely, albedo light, which cannot be collected by monofacial solar cells (*33*–*36*). In contrast, bifacial solar cell with double-sided light harvesting capacity can theoretically achieve higher energy yields than monofacial solar cells and hold great promise for improving power generation in actual applications. Figure 1A illustrates some application scenarios of bifacial solar cells. Now, commercialized bifacial silicon solar cells have shown great superiority in solar energy conversion. Notably, the thickness of the silicon layer is approximately 200 μm, which allows for strong light harvesting under both front and back illumination (*37*–*39*). In comparison, bifacial OSCs hold higher potential in some applications that need features like mechanical flexibility and light weight. The decisive difference between bifacial and monofacial solar cells is the rear transparent electrode (TE) that permits the propagation of albedo light into the active layer. However, it is well known that OSCs using rear TE are always transparent, implying severe optical loss and poor efficiency (left part of Fig. 1B) (*40*). The root cause is that the active layer is only about 100-nm thick, making it impossible for bifacial OSCs to effectively capture both front sunlight and albedo light. Reducing thickness to about 10 to 20 nm makes Ag films transparent while maintaining good conductivity, which enables OSCs the ability of albedo light harvesting. But the low refractive index and rough surface of ultrathin Ag film result in high reflection and absorption. Adopting optical coupling layers [like tellurium oxide (*41*, *42*), magnesium fluoride (*40*, *43*), molybdenum oxide (*44*, *45*), and zinc sulfide (ZnS) (*46*, *47*)] and introducing seed layers [like gold (Au) (*48*), aluminum (*49*), and self-assembly monolayers (*50*)] have shown to be effective strategies for pertinently mitigating optical loss of an ultrathin Ag rear electrode as mentioned. However, considerable parasitic absorption of ultrathin Ag film is still present due to nonideal film morphology and high extinction coefficient, thereby inducing optical loss of OSCs. Increasing the thickness of the active layer appears to be an effective approach to boost the light harvesting of bifacial OSCs. However, the thicker active layer delays the charge extraction and instead degrades the device performance of OSCs. Up to now, the state-of-the-art monofacial OSCs feature 500 nm active layer only obtained a PCE of 16%, which largely lags behinds the record PCE of more than 20% (*51*–*53*). Therefore, efficient bifacial OSCs have not been reported.

In this work, we proposed optically enhanced bifacial OSCs that feature efficient harvesting of both front sunlight and albedo light by synergistically regulating the optical and SP dynamics. Introduced asymmetrical optical transmission (AOT) array endows the rear electrode with strong reflection and antireflection for front and rear illumination, respectively, thus greatly improving the light harvesting of bifacial OSCs. The SP-induced energy loss at a transparent Ag electrode is then decreased by suppressing the excitation of SPs and transfer SPs to far-field radiation through SP-enhanced emission. At a conventional albedo of 0.2, the optimized bifacial OSCs feature low non-absorption loss and reduced parasitic absorption, resulting in outstanding PCE of 20.4%, which is higher than about 18% of the monofacial device. Outdoor testing and power generation calculation imply that the optimized bifacial OSCs can be more efficient in application scenarios with higher albedo factor.

## RESULTS

### Asymmetrical optical transmission array

The left part of Fig. 1B illustrates the schematic design and ray tracing diagram of optically enhanced bifacial OSCs. To suppress the transmission of sunlight and retain propagation of albedo light, a transparent pyramid array is set on the rear TE as an AOT. To reflect transmitted sunlight back to the active layer, the pyramid should be inclined at an angle of 45° according to Snell’s law. Under normal incidence conditions, two total internal reflections should occur within the pyramid. Optical simulations were firstly performed using finite-difference time-domain (FDTD) method to screen the transparent material of AOT. Three commercial materials including polydimethylsiloxane (PDMS), poly(methyl methacrylate) (PMMA), and polycarbonate (PC) were selected due to their high transparency in visible to near-infrared regions and varied refractive indices (fig. S1). Figure S2 plots the simulated reflection spectra of PDMS-, PMMA-, and PC-based AOT with a structural period of 40 μm. A flat glass surface is taken as the reference. Such flat surface has low reflection of around 5%, representing escaping of sunlight that causes severe optical loss. Regardless of the material chosen, the introduction of AOT greatly inhibits light escape. Notably, the reflectance of AOT gradually decreases with the wavelength. This is owing to the wavelength-dependent characteristics of refractive index of these materials. The AOT based on PDMS has the poorest asymmetrical optical transmission effect with an average reflectance of 83.7% (300 to 1000 nm). PDMS has a relatively low refractive index in the long wavelength region and cannot support twice total internal reflections in AOT. Conversely, a higher refractive index not only increases the reflectivity but also reduces the wavelength dependence of the reflection. As a result, PMMA- and PC-based AOTs obtained higher average reflectance of 95.0 and 97.3%, respectively. Figure S3 shows the average reflectance of the sample versus the angle of incidence. The advantages of PC over PMMA are more pronounced at higher angles of incidence. This facilitates wide-angle light collection for bifacial OSCs. To study the asymmetrical optical transmission of various structures, transmission spectra were also simulated with rear illumination (fig. S4). The adoption of 40-μm pyramids improves the average transmittance of 95.2% for flat surface to 98.7, 98.6, and 97.7% for AOTs based on PDMS, PMMA, and PC, respectively. Moreover, only a slight decrease in transmittance is observed while increasing the angle of incidence. The proven wide-angle antireflective effect suggests that AOTs can help bifacial OSCs achieve efficient collection of albedo light. Accordingly, PC was chosen as the medium to build AOT because of its superior asymmetrical optical transmission. To optimize the asymmetrical optical transmission effect, optical simulation of AOTs with different period was performed. Simulated reflection spectra in fig. S5A suggest that the light-blocking ability of AOT gradually decreases with reducing period of the pyramid array, especially at long wavelength region. Furthermore, the electric field distribution profiles of 5-, 20-, and 40-μm AOTs were also simulated (fig. S5, B to D). Normalized color bars and downward 800-nm light source were used. The periodic patterns at the edge represents the evanescent wave at the air side of the interface, which generated by the total internal reflection at PC/air interface. Strong light escaping is found at the tip of the pyramid. The electric field in air side of 5-μm AOT is stronger than the others, representing higher transmission. This result may be due to diffraction, since the structure size is close to the subwavelength scale. In contrast, AOTs with larger pyramids have suppressed electric field in air side. Therefore, it can be concluded that diffraction is rarely observed for AOT with large pyramids, and the asymmetrical optical transmission effect increases with increasing the period of pyramid. Taken into account of material cost and weight of AOT, the 40-μm pyramid was selected to enhance the light harvesting of bifacial OSCs.

**Fig. 2.**
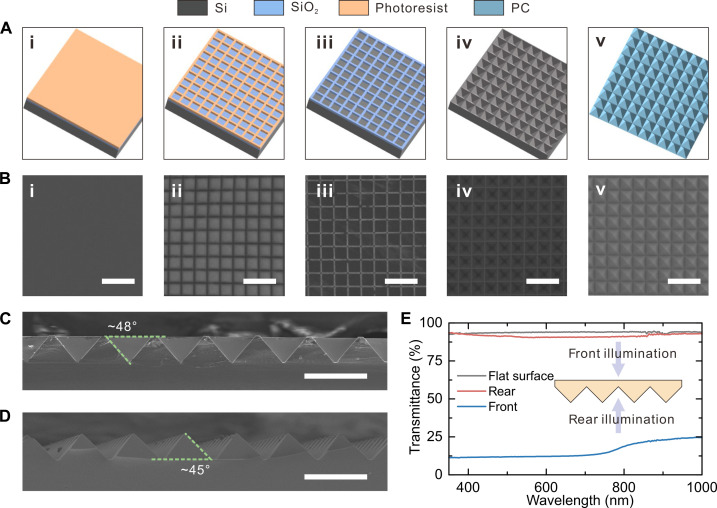
Fabrication and measurement of antitransmissive structures. (**A**) Schematic fabrication process and (**B**) corresponding SEM images of antitransmissive structures including (i) photoresist deposition on oxidized silicon wafer, (ii) ultraviolet exposure with shadow mask, (iii) oriented etching, (iv) photoresist removal, and (v) duplication. Scale bars, 100 μm. Cross-sectional SEM images of (**C**) prepared silicon mold and (**D**) duplicated structure on PC. Scale bars, 50 μm. (**E**) Transmission spectra of PC antitransmissive structure with front and rear illumination.

To obtain the PC-based AOT, a silicon mold with inverted pyramid array was first prepared by following the process as illustrated in Fig. 2A. The silicon wet anisotropic etching was adopted. The photoresist on the oxidized silicon wafer was patterned by the photolithography to form a square array with a period of 40 μm and a spacing of 5 μm. The uncured photoresist was dissolved in developer solution. After etching the exposed SiO_2_ with reaction ion etching and removing the residual photoresist with acetone, the square grid pattern was transferred to SiO_2_ layer and acted as a shadow mask. The obtained sample was then put into hot alkaline etching solution for anisotropic wet etching. The silicon mold was obtained by removing the SiO_2_ mask using hydrofluoric acid solution with a concentration of 10%. To ensure the conformal duplication of the pyramid structure, hydrophobic treatment was carried out by placing a silicon mold and 5 ml of chlorotrimethylsilane in a vacuumed chamber for 10 min. Last, the structure was transferred to PC by hot-imprinting technique. The processing details can be seen in Materials and Methods. Figure 2B shows the scanning electron microscope (SEM) images corresponding to each step in the fabrication process. The obtained SEM images have a good consistency with the schematic diagrams. After each process, there were no visible cracks or breaks in the silicon. The square shape remained unchanged and was completely transferred to the silicon wafer. A 40-μm period was maintained throughout the process, while the spacing was decreased from 5 to 3 μm. Reduced spacing is beneficial to enhance optical manipulation because the proportion of the flat area is reduced. In addition, cross-sectional SEM images of silicon molds and patterned PCs were characterized to check the tilt angle of the pyramids (Fig. 2, C and D). The anisotropic etching of silicon produces an inverted pyramid with a tilt angle of about 48°, whereas the tilt angle of the positive pyramid replicated on the PC film decreases to about 45° as the design requires. The decrease in the inclination of the pyramid stems from the shrinkage of PC after cooling (*54*).

The optical properties of prepared AOT were explored by measuring the transmission spectra. Notably, reflection spectrum under normal incidence is unavailable since the light source and detector cannot be simultaneously aligned in normal direction of the sample. As the results shown in Fig. 2E, under rear illumination, the transparency of the AOT is 91.7%, which is close to the 93.5% of flat glass. It is also found that the front light illumination is almost blocked by the AOT and greatly reduced the average transmittance to 15.4%. However, the measured asymmetric optical transmission effect is less effective than that estimated by optical simulations. Considering the duty cycle of AOT was set to 100% in simulation, we ascribe the difference to the presence of spacings in pyramidal structure. Therefore, silicon molds based on masks with reduced spacing sizes were fabricated to improve the asymmetric optical transmission. Figure S6 (A and B) displays top-view and cross-sectional SEM images of silicon molds processed using shadow masks with widths of 4 and 3 μm. After etching, a period of 40 μm was remained with the regardless of width of shadow mask. A shadow mask with a width of 4 μm produces a spacing reduction of 1.5 μm, while a shadow mask with a width of 3 μm markedly increases the spacing to more than 12 μm. Cross-sectional SEM images imply that the 3-μm-wide SiO_2_ cannot withstand hot alkaline etching and disappears after a period of etching, leading to deformation of the pyramidal structure. Accordingly, as depicted by fig. S6C, the asymmetric optical transmission was greatly weakened, resulting in a high average front transmittance of 39.9%. For the 4-μm-wide SiO_2_-processed AOT, a reduced average front transmittance of 13.4% was obtained with a simultaneously increased average rear transmittance of 92.5%.

### SP dynamics in asymmetrical transmission electrode

**Fig. 3.**
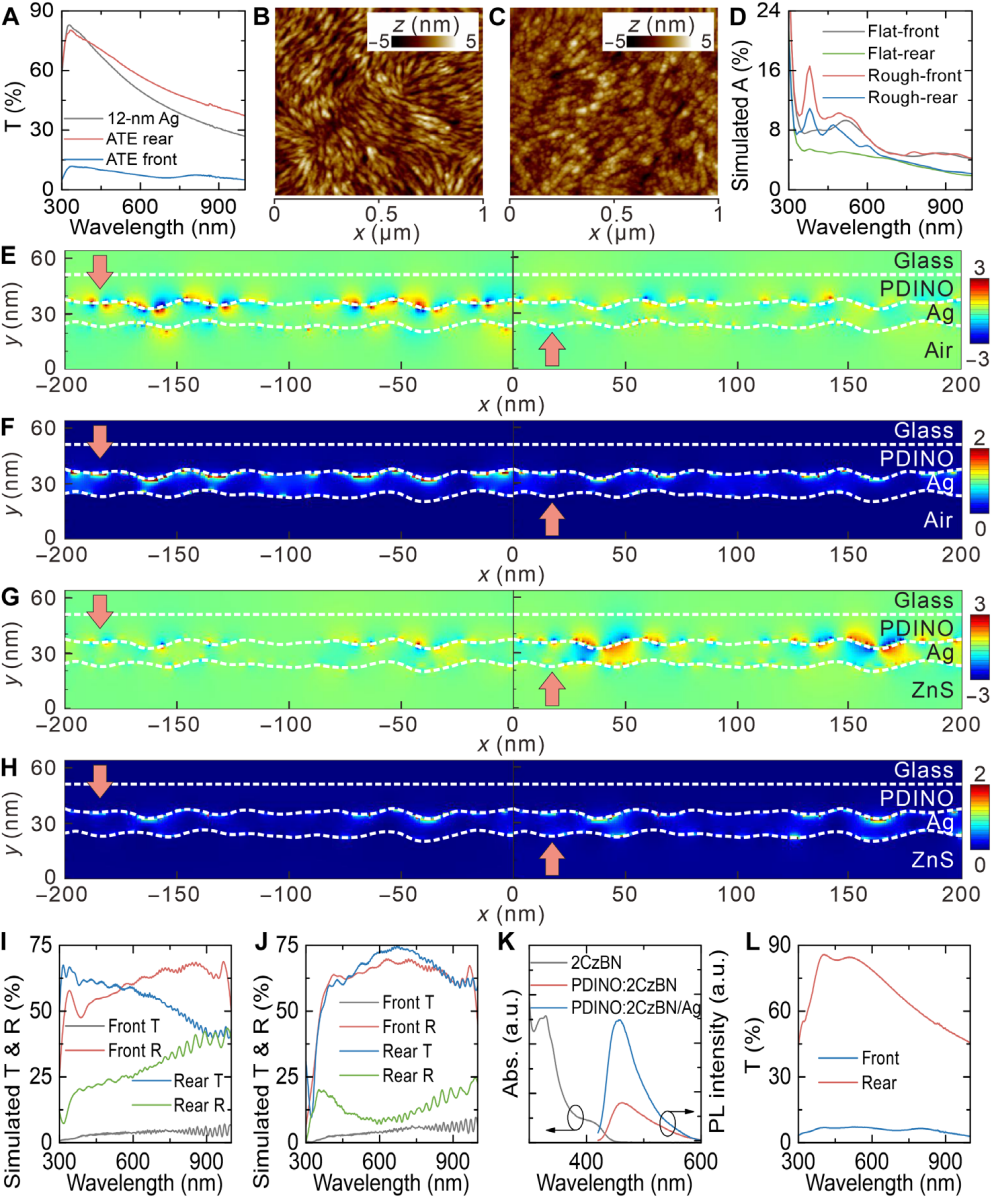
Optoelectronic properties of ATEs. (**A**) Transmission spectra of 12-nm Ag and ATEs under different illumination direction. AFM height images of (**B**) PDINO and (**C**) PDINO/12-nm Ag. (**D**) Simulated absorption spectra of 12-nm Ag with flat and rough surfaces. Simulated (**E** and **G**) *E_y_* field and (**F** and **H**) absorption distribution profiles of rough Ag film [(E) and (F)] without and [(G) and (H)] with optical coupling layer. The arrows represent the direction of 380-nm illumination. Simulated transmission and reflection spectra of ATEs (**I**) without and (**J**) with ZnS film. (**K**) Absorption spectrum of 2CzBN and PL spectra of PDINO:2CzBN and PDINO:2CzBN/12-nm Ag. (**L**) Transmission spectra of SP-ATE under different illumination direction. T., transmittance; A., absorbance; R., reflectance; a.u., arbitrary units.

The optimized AOT was integrated with a transparent silver (Ag) electrode with a thickness of 12 nm to explore its ability of optical manipulation in bifacial OSCs. Notably, 0.5-nm Au was adopted as seed layer to improve the surface morphology of ultrathin Ag film. The AOT-integrated Ag electrode (asymmetrical transmission electrode) is denoted as ATE. Figure 3A depicts the transmission spectra of 12-nm Ag and ATE under rear or front illumination. It is well known that the transmittance of systems consisting of planar films is independent of the direction of light, so only one transmission spectrum of 12-nm Ag is shown here. The 12-nm Ag exhibits a transmission peak at 330 nm and features gradually decreased transmittance with increasing wavelength. A low transmittance of below 25% at 900 nm was observed. While high reflectivity helps collect light from the front more efficiently, it can also impede the propagation of light from the rear. Besides, according to the reflection and absorption (1-R-T) spectra, a portion of light is absorbed by 12-nm Ag (fig. S7). As for ATE, the front transmittance across the spectrum is as low as about 10%. The escaping of front illumination is supposed to be effectively suppressed with the introduction of AOT. However, the reflectivity of ATE may not be ideal considering the considerable parasitic absorption of 12-nm Ag as demonstrated. The transmission spectrum of ATE under rear illumination exhibits similar trend as that of 12-nm Ag but with higher transmittance in the spectral region from visible to near infrared. The antireflection effect of AOT may be responsible for the result. The relatively low rear transmittance of the ATE is not enough to make it a good TE. As revealed by fig. S7, the low rear transmission of ATE stems from the reflection and absorption of 12-nm Ag.

To understand the optical dynamics in ATE, optical simulations based on the FDTD method were performed. The morphology of Ag film was characterized to provide more accurate modeling in the simulation setup. Figure 3B is the atomic force microscopy (AFM) height image of glass/poly (3,4-ethylenedioxythiophene) polystyrene sulfonate (PEDOT:PSS)/active layer/N,N′-bis(N,N-dimethylpropan-1-amine oxide)perylene-3,4,9,10-tetracarboxylic diimide (PDINO), showing the fibrous morphology of the bottom surface of the Ag film. While the AFM height images of active layer in fig. S8 reveals that the phase separation of active layer results in bumps. Therefore, according to related reports, the fibrous morphology is considered to be the result of PDINO aggregation (*55*, *56*). The AFM height image of 12-nm Ag film is displayed in Fig. 3C. Basically, with the help of Au seed layer, Ag deposited on PDINO grows along the nanofibers and forms a continuous film. Comparisons between AFM images suggest that the upper surface of Ag film is conformal to that of PDINO. Notably, Ag still tends to form in nanoparticle with diameter of about 10 nm. A reduced root mean square (RMS) of 1.2 nm is derived from the AFM image of 12-nm Ag. Figure 3D shows the simulated absorption spectra of 12-nm Ag with flat and rough surfaces. To simulate the real surface morphology, the RMS values of bottom and top surfaces of Ag film were set to 1.6 and 1.2 nm (fig. S9), respectively, based on the results obtained from the AFM images. For ideal flat Ag film, the absorption is originated from the intrinsic absorption of Ag, which is related to the optical admittance matching conditions. Under front illumination, i.e., light travels from optically denser medium (PDINO) to optically rarer medium (air), flat Ag film has an absorption around 8% at the spectral region below 600 nm. While the reversed optical path shows a weaker optical loss in flat Ag film, producing an absorption of below 5%. In contrast, the bump surface generates additional optical loss of the transparent Ag film. The simulated absorption of rough Ag film exhibits characteristic absorption peaks at 380 nm and around 500 nm, yielding a highest absorption of over 16%.

Electric field distribution profiles of ATEs were simulated to reveal the origin of parasitic absorption of rough Ag film. Figure 3E presents the electric field along *y* axis (*E_y_*) of rough Ag film under front and rear illumination. The observed symmetric patterns represent the presence of SPs at the interface. In comparison, stronger SPs can be excited under front illumination. Consequently, relatively higher absorption was found at corresponding bumps as shown in the simulated absorption profiles (Fig. 3F). The presence of SPs was further confirmed by investigating the *E_y_* distribution profile of flat Ag film, where no notable *E_y_* signal was found (fig. S10A). Therefore, the intrinsic absorption of Ag generates a lower optical absorption as indicated by fig. S10B. Considering that the optical constant mismatch relates to both the excitation of SP and the propagation of light, the outer medium was changed to minimize the parasitic absorption and maximize the transparency of Ag film. By capping an optically denser medium of ZnS on Ag film, the optical dynamic in Ag film is obviously changed. Under front illumination, both SPs at the upper and lower surfaces of Ag is suppressed (Fig. 3G). Notably, the rough Ag film has bumps with a diameter (*d*) of around 10 nm and a thickness (*h*) of 12 nm. Both the equations of *d* ≪ λ and $h\ll \delta =\frac{\lambda }{4\pi \sqrt{\varepsilon_{m}}}$ are fulfilled, where λ is the wavelength, δ is the skin depth of Ag, and ε_m_ is the dielectric constant of Ag. Therefore, Rayleigh (or quasi-static) approximation can be applied to the analysis of the SP dynamic in rough Ag film. If we classify the excited SP to localized SP resonance (LSPR) and consider the rough Ag film as closely packed Ag nanoparticles, the outer electric potential (Ф) should follow the equation of $\Phi =-E_{0}r\text{cos}\theta +\frac{p\cdot r}{4\pi \varepsilon_{0}\varepsilon_{d}r^{3}}$, where dipole moment (***p***) can be derived from $p=\alpha E_{0}=4\pi \varepsilon_{0}\varepsilon_{d}d^{3}\frac{\varepsilon_{m}-\varepsilon_{d}}{\varepsilon_{m}+2\varepsilon_{d}}E_{0}$. In the equations, α is the polarizability, ε_d_ is the dielectric constant of dielectric film, **r** is the unit vector, and ***E***_0_ is the external field. According to references, Ag has a ε_m_ = −4 + i0.5 at around 380 nm. Considering the resonance condition of ε_m_ + 2ε_d_ = 0, the LSPR is stronger at the PDINO side because PDINO has a dielectric constant of around 2.5. By replacing the outer medium from air to ZnS, the high ε_d_ of 6 produces an increased ***p***, which represents a stronger LSPR. However, as displayed in fig. 3G, SPs at both upper and lower interfaces are suppressed. The opposite conclusion suggests that the generated SPs cannot all be categorized to LSPR.

Another type of SP is the SP polariton (SPP), which is a collective oscillation of electrons as they propagate along the interface between a metal and a dielectric film. It is well known that the dispersion of SPP on a smooth surface is given by $k_{\text{spp}}=k_{0}\sqrt{\frac{\varepsilon_{m}\varepsilon_{d}}{\varepsilon_{m}+\varepsilon_{d}}}$, where **k**_spp_ and **k**_0_ is the wave vector of SPPs and photons, respectively. Since the SPP dispersion curve does not overlap with the photon dispersion curve of the dielectric material, the SPPs cannot be excited directly with illumination from adjacent dielectric medium. Whereas, on rough surfaces, the resonance condition can be realized because the diffracted components with all wave vectors are present in the near-field region. Notably, the light-to-SPP coupling efficiency on rough surface is relatively low compared to the case where prisms or diffraction gratings are used. As the SPPs propagating along metal surface suffer from severe nonradiative damping, optical loss is commonly observed, which may be the origin of high absorption of rough Ag film. Therefore, according to the equation, the excitation of SPPs can be suppressed by adopting dielectric medium with ε_d_ larger than ε_m_. As it has been revealed by Fig. 3 (E to H), the front illumination is partially transformed to nonradiative SPPs and absorbed at the structure of PDINO/rough Ag/air. A closer inspection of Fig. 3E suggests that the *E_y_* distribution at Ag/air interface is closely related to the SPs at PDINO/Ag interface. Unlike the symmetrical and heterochromatic patterns found on the upper surface, the patterns on the lower surface is asymmetric and has the same color as the adjacent SP patterns at upper surface. Because the thickness of Ag is much smaller than the skin depth of Ag, we speculate that there are strong electrical interactions between the upper and lower surfaces of Ag film. The interaction between SPPs and LSPRs exists because the feature size of bumps is able to excite both SPs. This mechanism leads to resonant LSPR-to-SPP coupling and vice versa transformation, thereby confining light at bumps. As the distance between bumps is much smaller than the propagation length of SPP, we assume that the excited SPPs are quickly transferred to LSPR. This is confirmed by the distribution of *E_y_* on Ag, which does not have a continuous pattern with periodic varied intensity. The interplay of LSPR and SPP generates the featured distribution of *E_y_* shown in Fig. 3E. Therefore, by replacing air with ZnS, the excitation of SPP is suppressed, and the coupling between SPs is weakened, which generates lower optical loss. According to the simulated transmission and reflection spectra of ATEs with and without ZnS plotted in Fig. 3 (I and J), the front reflection from ATE is increased owing to the reduced absorption. In our work, both simulated and measured results indicate that the optical modulation effect of AOT and ZnS reduced the optical reflectance of the transparent rear electrode, yielding bifacial OSCs with enhanced light harvesting on both sides. Therefore, the related researches and our experimental data demonstrate that the optical modeling strategy in this work features high feasibility and reproducibility (*40*, *42*, *46*).

An unexpected result is the appearance of enhanced SPs at the PDINO/Ag interface after introducing ZnS films (Fig. 3G). Even if the rear transmission is increased in a broad spectral region, the rear reflection is simultaneously decreased due to the enhanced optical loss (Fig. 3, I and J, and fig. S11). We ascribe the results to the fact that ZnS film changes the wave vector (**k** = 2π/λ = 2π*n*/λ_0_, where λ_0_ is the wavelength in vacuum) in adjacent medium. Accordingly, with the SPP excitation at Ag/ZnS interface restrained, the generation of SPs at PDINO/Ag interface becomes easier due to the smaller mismatch in dispersion curves. Although the boosted SP generation under rear illumination contributes to additional parasitic absorption, the ZnS-integrated ATE exhibits good asymmetric optical transmission. Figure S11 reveals the presence of considerable optical loss in ZnS-integrated ATE. To recycle the SP energy, a SP-enhanced emission configuration was further adopted here. An organic emitter (2CzBN) was doped in PDINO and acted as extraction channel for SP energy. As the absorption spectrum of pure 2CzBN film shown in Fig. 3K, 2CzBN absorbs light with a wavelength below 440 nm. The photoluminescence (PL) spectra of PDINO:2CzBN and PDINO:2CzBN/12-nm Ag were also characterized (Fig. 3K). The absorption and PL spectra of 2CzBN overlap well with the broadband absorption of ATE, which facilitates the energy transfer between SPs and 2CzBN. As expected, the PL intensity of PDINO:2CzBN was increased by more than three times due to the SP-enhanced emission. Considering that the PDINO film features a small thickness of about 10 nm, strong Förster resonance energy transfer between 2CzBN and SPs is expected. There is competition between SP-enhanced nonradiative decay that transfer energy from the emitter to the SP and SP-enhanced radiative decay that transfer energy from the SP to the emitter. The enhanced PL intensity suggests the SP-enhanced nonradiative decay dominates the energy transfer. The quantum yield of 2CzBN was largely improved, which benefits the SP energy recycling. Furthermore, SPs provide a strong confinement of electromagnetic field. The excitation rate of 2CzBN is thus increased through Purcell effect at the absorption wavelength. The ATE with SP regulation using ZnS and 2CzBN, denoted as SP-ATE, exhibits an obviously enhanced asymmetric optical transmission effect compared to the pristine ATEs (Fig. 3L). The optimized ATE achieves a low front transmittance of below 7% and a high rear transmittance of up to 86%.

### Photovoltaic performance of bifacial OSCs

**Fig. 4.**
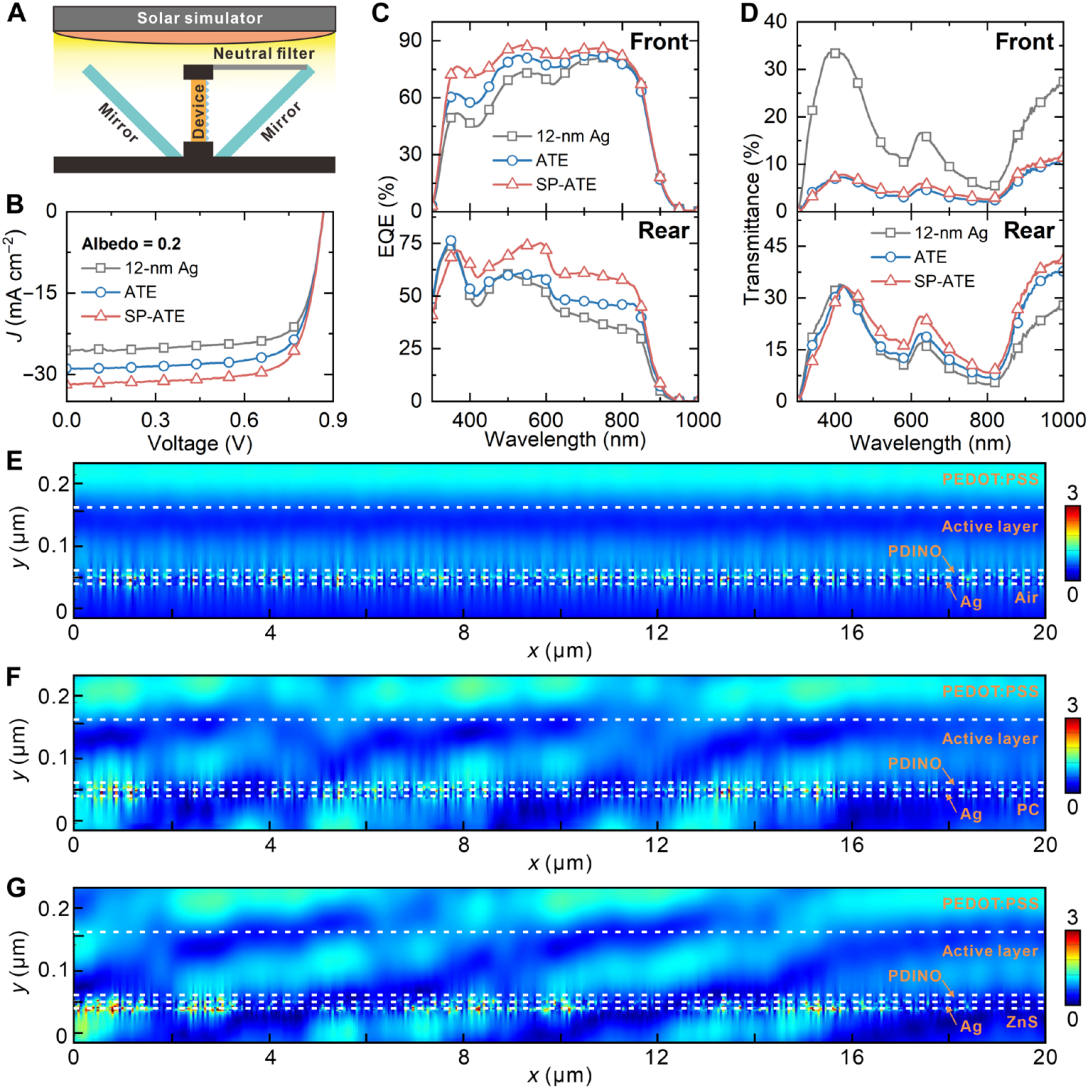
Photovoltaic performance of bifacial OSCs. (**A**) Schematic diagram of measurement system. (**B**) *J-V* curves of bifacial OSCs under the AM 1.5G illumination with a power intensity of 100 mW cm^−2^. The albedo factor is 0.2. (**C**) EQE spectra and (**D**) transmission spectra of bifacial OSCs under front (top) and rear (bottom) illumination. Simulated electric field distribution profiles of bifacial OSCs based on (**E**) 12-nm Ag, (**F**) ATE, and (**G**) SP-ATE. Front and rear light sources with a power intensity ratio of 1:0.2 and a wavelength of 500 nm were applied.

To validate the light-harvesting enhancement effect of the ATEs on the device performance, bifacial OSCs with the device architecture of glass/indium tin oxide (ITO)/PEDOT:PSS/D18:Y6:Y6-1O/PDINO/TE were fabricated (details can be seen in Materials and Methods). It should be mentioned that the monofacial devices using 100-nm-thick Ag film were taken as a reference. A good PCE of 17.96% with an open-circuit voltage (*V*_OC_) of 0.88 V, a short-circuit current density (*J*_SC_) of 26.78 mA cm^−2^, and a fill factor (FF) of 76.20% was obtained for a monofacial device (Table 1 and fig. S12). The obtained PCE is comparable to the reported values of OSCs with the same active layer (*57*). The thickness of the Ag electrode was decreased to 12 nm as designed to prepare bifacial OSCs. The sheet resistance of the rear electrode has been measured to explore the conductivity. The 12-nm Ag film shows a sheet resistance of 7.2 ohm sq^−1^, which represents good charge collection ability. Meanwhile, according to related reports, the thickness of Ag film has negligible influence on work function (*58*, *59*). Therefore, as the energy level diagram depicted in fig. S13, the cascade alignment of energy level structure is beneficial for charge transfer and extraction. We thus conclude that the transparent rear electrode features good electrical characteristics. For the characterization of bifacial devices, a testing criterion proposed by International Electrotechnical Commission (IEC TS 60904-1-2) was used here for standardized indoor characterizations to precisely evaluate the efficiency of bifacial OSCs (*33*–*35*). Figure 4A displays the schematic diagram setup for measuring bifacial OSCs under bifacial illumination consisting of a solar simulator, a filter, and two mirrors with 45° inclination angle to device. A neutral optical filter with transmittance of 20% was used to adjust the power density of rear illumination to 20 mW cm^−2^, simulating the practical albedo scenario with the most widely used albedo factor of 0.2. Figure 4B plots the current density-voltage (*J-V*) characteristics of bifacial OSCs. The detailed photovoltaic parameters inferred from the *J-V* curves are summarized in Table 1. The control bifacial OSC with the 12-nm Ag electrode has an inferior *V*_OC_ of 0.87 V, a *J*_SC_ of 25.58 mA cm^−2^, and a FF of 74.76%, yielding a PCE of 16.60%. Compared to a monofacial device, the relatively lower *J*_SC_ and FF owe to the light escaping from the rear electrode and higher sheet resistance of 12-nm Ag film, respectively. On the contrary, the introduction of ATE in bifacial OSCs leads to an enhanced *J*_SC_ of 28.87 mA cm^−2^, attributing to the synergistic effect of the asymmetric optical transmission and antireflection of ATE. With almost the same *V*_OC_ and FF, the ATE-based device obtained an increased PCE of 18.67%, which is higher than the monofacial device. The device performance was further enhanced by using SP regulation strategies. Optimization on SP-ATE–based bifacial OSCs was also carried out by varying donor/acceptor (D/A) ratio, active layer thickness, and additives. The detailed photovoltaic parameters are summarized in tables S3 to S5. Changes in the D/A ratio have a notable impact on *J*_SC_ and FF owing to different phase morphology. The device with D/A ratio of 1:1.6 achieved the balance between light harvesting, exciton dissociation, and charge extraction, yielding an outstanding PCE over 20%. As the cross-sectional SEM image shown in fig. S14, the thickness of active layer in bifacial OSCs is about 103 nm, which is similar to the reported values. In addition, bifacial OSCs with different active layer thickness were characterized. As the device performance summarized in table S4, the thicker active layer absorbs more sunlight and obtains higher photocurrent. However, owing to the poor carrier mobility of the active layer, the FF is reduced. In contrast, charge recombination is suppressed in the device with a thinner active layer, but the photocurrent is simultaneously decreased due to insufficient light harvesting. With the tradeoff between light harvesting and charge extraction, the device with 103-nm active layer obtained the best PCE. In addition, the additive was changed to optimize device performance. It is found that the 1-chloronaphthalene-based device has the highest FF but a relative lower photocurrent (*60*). On the basis of these optimizations, the *J*_SC_ of SP-ATE–based device was increased to 31.83 mA cm^−2^ with *V*_OC_ and FF maintain almost unchanged, producing a high PCE of 20.44%. This is the highest reported PCE for OSCs.

**Table 1.** Photovoltaic performance of bifacial and monofacial OSCs. Photovoltaic parameters for monofacial and various bifacial OSCs under 100 mW cm^−2^ AM 1.5G illumination. The albedo factor is 0.2. The average PCE (PCE_ave_) were obtained from 16 devices.

Rear electrode	*V*_OC_ (V)	*J*_SC_ (mA cm^−2^)	FF (%)	PCE (%)	PCE_ave_ (%)
Opaque	0.88	26.78	76.20	17.98	17.76
12-nm Ag	0.87	25.58	74.76	16.60	16.32
ATE	0.87	28.87	74.60	18.67	18.28
SP-ATE	0.87	31.83	74.03	20.44	20.05

Photovoltaic characteristics of various bifacial OSCs under solely front or rear illumination are listed in table S1. The reference device exhibits PCEs of 15.08 and 9.08% under front and rear illumination, respectively. The poor device performance is ascribed to the substantial optical losses of 12-nm Ag as demonstrated. After introducing ATE, front and rear device efficiencies were increased to 16.03 and 9.70% owing to the improved *J*_SC_. Further increase in efficiencies was achieved with the introduction of SP-ATE which largely reduces optical loss. The remarkable PCEs of 17.57 and 12.75% are obtained for optimized device under front and rear illumination, respectively. According to IEC TS 60904-1-2, the equivalent efficiencies of 12-nm Ag-, ATE-, and SP-ATE–based bifacial OSCs under the albedo of 0.2 are 16.89, 18.55, and 20.12%, respectively. The equivalent efficiencies demonstrate a good conformance with the measured values in Table 1, indicating the superiority of SP-ATE in bifacial OSCs. We also tested device performance of bifacial OSCs in the National Center of Supervision & Inspection on Solar Photovoltaic Products Quality of China (CPVT). The certified PCEs of bifacial OSCs are 17.52 and 12.71% under front and rear illumination, respectively (fig. S15). The verified results are in good accordance with the measured values in our labs, verifying the outstanding albedo-collecting ability of SP-ATE–based OSCs. To evaluate the performance of bifacial OSCs as transparent OSCs, average visible transmittance (AVT) values of various bifacial OSCs were calculated and summarized in table S2. The AVT values of 12-nm Ag-based bifacial OSC under front and rear illumination are 13.58 and 13.78%, respectively. Under front illumination, most of the incident light is absorbed by the active layer, while under rear illumination, reflection from the rear electrode is the main cause of optical loss. Therefore, the LUE of 12-nm Ag-based bifacial OSC is much lower under rear illumination. After introducing ATE, the front AVT of bifacial OSC decreased to 3.70% due to the light deflecting effect of AOT, yielding an ultralow LUE of 0.61%. Meanwhile, the antireflective effect of AOT under rear illumination causes a relatively higher AVT and LUE. The reduced parasitic absorption in SP-ATE–based device improved AVT under both front and rear illumination. A highest rear LUE of 2.48% was obtained. Accordingly, SP-ATE–based devices may act as semitransparent OSCs regarding their advantages on high output power and acceptable LUE.

To study the adhesion between AOT and the rear electrode, adhesion measurement was carried out by using peeling mode of universal testing machine. As depicted in fig. S16, the adhesion force between AOT and the Ag electrode is about 0.44 N, which could guarantee the regular operation of bifacial OSCs (*61*). Furthermore, thermal stability of bifacial OSCs was also explored by placing a device on a hot plate at 100°C and testing at a certain time interval (fig. S17). It is found that PCEs can still maintain about 99% of its initial values after 4 min of heating. As the hot-pressing process needs only 2 min of 100°C heating, we derived that the hot-pressing process has negligible influence on device performance.

External quantum efficiency (EQE) spectra were measured to elucidate the differences in *J*_SC_ between devices. It is found that the EQE response performed differently under front and rear illumination as plotted in Fig. 4C. Under front illumination, the integrated *J*_SC_ values derived from the EQE spectra of 12-nm Ag-, ATE-, and SP-ATE–based bifacial OSCs are 22.8, 24.4, and 26.2 mA cm^−2^, respectively. Refer to the transmission spectra of rear electrodes and OSCs illustrated in Figs. 3A and 4D, the relatively low EQE of the 12-nm Ag-based device stems from obvious transmission-induced light escaping from the rear electrode. In contrast, ATE decreased the average front transmittance of OSCs from 16.7 to 4.9%, effectively blocking transmission-induced optical loss, especially at short wavelength region. Accordingly, the EQE of the ATE-based device under front illumination is improved compared to the 12-nm Ag-based device. Notably, as demonstrated in Fig. 3L, SPs excited on Ag surfaces cause parasitic absorption that limits the photocurrent of OSCs, while the proposed SP-ATE suppresses parasitic absorption losses and shows improved asymmetric optical transmission effect. Therefore, although the average front transmittance of OSCs increased to 5.6% due to boosted transmission-induced light escaping from the spacings between pyramids, the SP regulation strategy further improved EQE due to the reduction of parasitic absorption in the 12-nm Ag film [top of Fig. 4 (C and D)]. As for the device under rear illumination, 12-nm Ag-, ATE-, and SP-ATE–based bifacial OSCs obtained integrated *J*_SC_ of 14.6, 16.6, and 20.1 mA cm^−2^, respectively. Taking into account the albedo factor of 0.2, the calculated *J*_SC_ values are within 5% error compared to the measured results. As the high reflectance of the rear electrode is beneficial to the harvesting of front illumination but is detrimental to the harvesting of rear illumination, the 12-nm Ag-based device has a low EQE value of around 35% under 800-nm rear illumination (bottom of Fig. 4C). Since the transmittance of 12-nm Ag film decreases with increasing wavelength, the EQE also shows the same decreasing trend (Fig. 3A). The ATE has limited influence on rear EQE spectrum since the normal incident light is deflected by AOT, which may increase the reflection from Ag film. The interplay of antireflective effect of AOT and reflection of Ag film results in the variation of rear EQE spectrum. After introducing SP-ATE, EQE response exhibits an impressive broadband improvement within the spectral region beyond 365 nm compared to the 12-nm Ag-based device. The decreased EQE at the ultraviolet region stems from the absorption of ZnS. As indicated in Fig. 3L, because of the largely suppressed reflection and reduced parasitic absorption of Ag film, rear illumination is effectively propagated through SP-ATE and then absorbed by active layer. Therefore, the SP-ATE–based device obtained an improved EQE spectrum under rear illumination.

Given the possibility of thin-film interference in the device, it is not appropriate to understand the optical dynamics by simply studying the optical properties of the rear electrode. Therefore, the simulations of electric field (|*E*|) distribution in bifacial OSCs were carried out. Front and rear light sources were simultaneously applied with a power intensity ratio of 1:0.2 and a wavelength of 500 nm. A 40-μm pyramid is symmetric about *y* axis. Figure 4 (E to G) illustrates the electric field distribution profiles in OSCs based on 12-nm Ag, ATE, and SP-ATE, respectively. The interface between active layer and PDINO was set to be planar to minimize the computational effort, since SPs cannot be excited at this interface and have negligible influence on electric field distribution. For the 12-nm Ag-based device, dual-side illumination generates planar pattern along *x* axis. The SPs excited on Ag obviously affect the near-field distribution of electric field, endowing the electric field pattern near Ag with random variations. We usually assume that SPs promote OSC absorption through near-field scattering. However, in this case, the small size of the bumps on the Ag film leads to a different result. Considering that the scattering factor (η_sca_) and absorption factor (η_abs_) of LSPR are given by $\eta_{\text{sca}}=\frac{k^{4}}{6\pi \varepsilon_{0}^{2}}|\alpha |$ and $\eta_{\text{abs}}=\frac{k}{\epsilon_{0}}\text{Im}\;[\alpha ]$, respectively. For bumps smaller than 10 nm in diameter, scattering is almost nonexistent, and absorption dominates the light-matter interaction. This is also responsible for the low haze of transparent Ag film. Therefore, the excited SPs on Ag film barely contributes to the light harvesting of OSCs. Because of the rear light deflection at the AOT, the interference in the OSC is affected, causing the pattern to tilt in the −*x* direction. On the basis of the enhanced electric field around the *y* axis, we find that the AOT also acts as a concentrator. The SP excitation near *y* axis is therefore boosted. This phenomenon is more obvious in the SP-ATE–based device. These results suggest that, in ATE-based OSCs, the confined electromagnetic field generated by SP is stronger due to the tilted interference. This is beneficial to the SP-enhanced emission that further reduces SP energy loss.

**Fig. 5.**
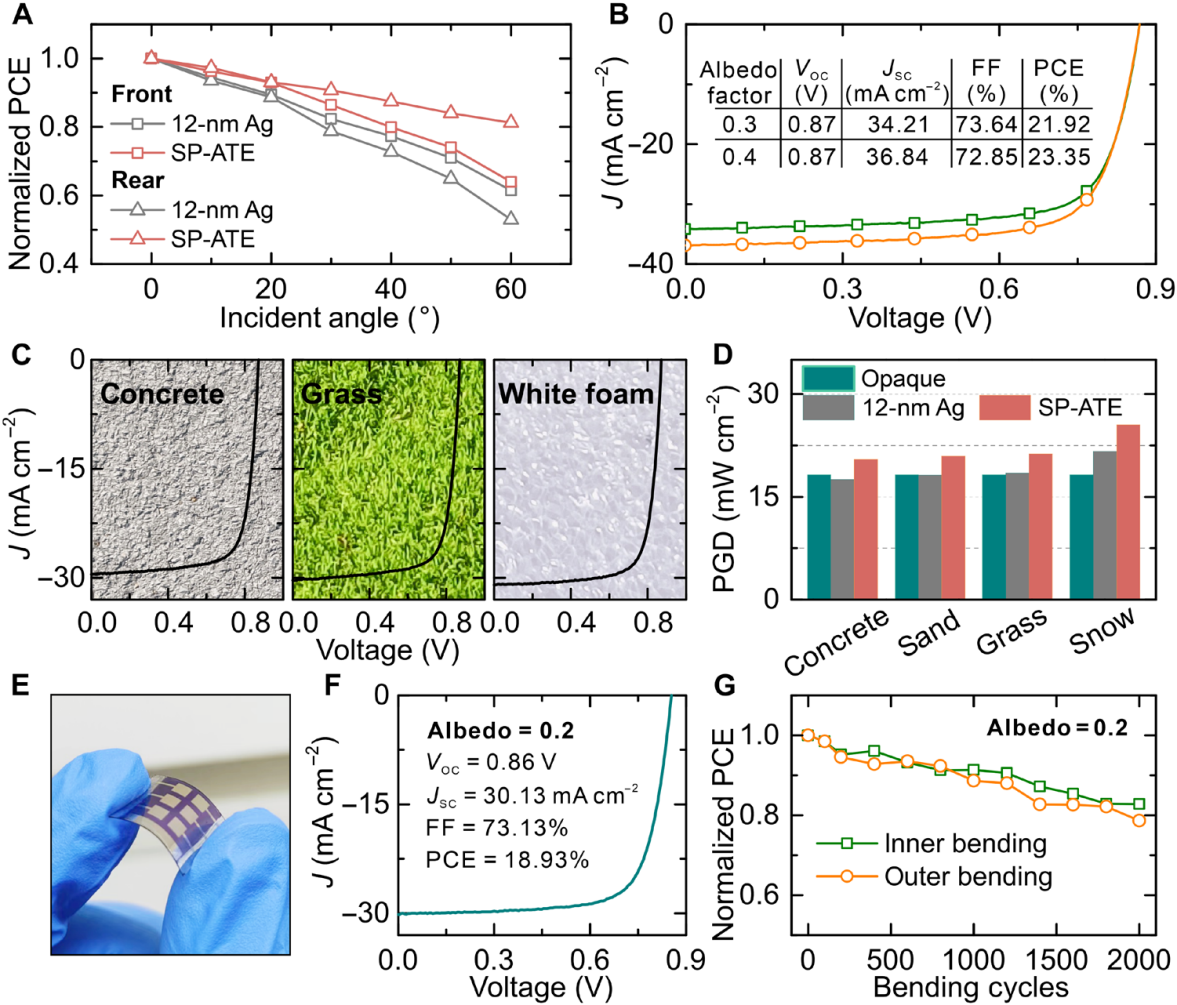
Outdoor testing and flexible application of bifacial OSCs. (**A**) Angle-dependent efficiency of 12-nm Ag- and SP-ATE–based OSCs under different illumination direction. (**B**) *J-V* curves of SP-ATE–based OSCs with increased albedo factor. (**C**) *J-V* curves of SP-ATE–based OSCs measured in real scenarios with concrete, grass, and white foam as background, illustrated with corresponding background photos. (**D**) Power generation analysis of monofacial OSC, 12-nm Ag-based bifacial OSC, and SP-ATE–based bifacial OSC. (**E**) Photograph, (**F**) *J-V* curve, and (**G**) bending test of flexible SP-ATE–based OSC.

To investigate the light harvesting capability of ATE-based OSCs over a wide range of angles, device efficiencies were recorded for different irradiation angles. As depicted in Fig. 5A, it is obvious that the efficiency of the 12-nm Ag-based device decreases rapidly, remaining only 61.4 and 52.9% of their initial values under front and rear illumination, respectively, with the increasing illumination angles to 60°. The large reflection of the glass/ITO and Ag electrodes can ascribe to the efficiency decay. Under the same condition, front and rear PCEs of SP-ATE–based device obtained 63.9 and 81.3% of their initial values. No large difference was found for the devices under front illumination. In contrast, the light harvesting of bifacial OSC is greatly improved by introducing SP-ATE. This is owing to the antireflective effect of pyramids. Meanwhile, photovoltaic characteristics of SP-ATE–based OSC under different albedo were measured to explore the performance of SP-ATE. Different albedo was realized by changing the neutral filters regarding the testing standard IEC TS 60904-1-2. As the *J-V* curves plotted in Fig. 5B, outstanding efficiency of 21.92% was achieved under the albedo of 0.3. The PCE as further increased up to 23.35% under the albedo of 0.4, which implies the distinctive superiority of the SP-ATE–integrated bifacial OSCs. Meanwhile, the outdoor testing of device performance in real application scenarios was performed to demonstrate the superiorities of SP-ATE. Figure 5C shows *J-V* curves of SP-ATE–based OSCs under noon sun irradiation with background changing sequentially from concrete to grass and white foam. As the concrete, grass, and white foam have sequentially higher albedo factor, the photocurrent of SP-ATE–based OSCs was gradually increased. SP-ATE–based bifacial OSCs display the promising prospects in improving power generation efficiency by reinforcing the light-harvesting ability of sunlight. We strengthened the comparison between monofacial and bifacial OSCs with simulated power generation density (PGD) to illustrate the benefits of AOT in albedo light harvesting using SunSolve Power. Interface materials and device structure were properly selected and designed to simulate the real situation. Different rear illumination situations with increasing albedos were applied, that is, concrete, sand, grass, and snow. As shown in Fig. 5D, the PGD of 12-nm Ag-based bifacial devices is inferior to the monofacial devices until the albedo is up to 0.4. It is found that a high PGD of 21.67% is obtained under a senior albedo (snow), even the 12-nm Ag-based devices have considerable optical loss. Improvements have been achieved after introducing SP-ATE. Notably, the efficiency has breached the limit of 20% under the common albedo (concrete). An impressive PGD of about 21% is obtained under sand and grass, indicating the unrivaled advantages of SP-ATE–based bifacial OSCs in practical applications. Furthermore, there is a sparkling PGD of 25.52% when the background is changed to snow. These results suggest that the bifacial OSCs with the integration of SP-ATE will be appealing for solar energy harvesting in the future.

In addition, the application of SP-ATE in flexible OSCs was explored. The fabrication process of flexible OSCs is described in Materials and Methods, which is finely optimized in our previous work. Figure 5E displays the photograph of the flexible device. *J-V* measurement was performed to examine the compatibility between SP-ATE and flexible devices. As depicted in Fig. 5F, the optimized flexible device obtained an impressive efficiency of 18.93%, which is the highest reported value to date. This result benefits from the superior optical modulation of SP-ATE, which obviously increases the photocurrent. It is worth noticing that mechanical stability was also evaluated under front and rear bending during the bending tests at a bending radius of 5 mm (Fig. 5G). SP-ATE–based flexible OSCs exhibited an excellent bending stability after 2000 cycles continuous bending, remaining about 80% of the initial PCE. The outer bending causes faster efficiency decay because of the large strain applied on the functional layers. The favorable bending stability demonstrates the compatibility and stability of AOT and Ag film.

The minimum sustainable price (MSP; ¥/W_p_) is a critical metric that determines the value of module sales for a photovoltaic (PV) manufacturer’s sustainable operations, which can be used in the cost analysis of photovoltaic module. According to related report, for monofacial OSCs with PCE of 17.96%, the MSP is about 1.71 ¥/W_p_, considering the difference of active layer from the literatures (*62*). Notably, the MSP of bifacial devices can be reduced to 1.53 ¥/W_p_ under the same condition, which is mainly credited to the efficiency improvement and closer to the optimally predicted MSP of 0.97 ¥/W_p_ for industrial scenario beyond 2025 (*63*). Take into account the cost of AOT, the final MSP of SP-ATE–based bifacial OSCs is estimated to 1.61 ¥/W_p_. Meanwhile, AOT can act as the encapsulation layer for bifacial OSCs, which is instrumental to device stability (*64*). Such the potential advantages in MSP and stability achieved by SP-ATE make it a promising strategy to improve the economic viability of OSCs.

## DISCUSSION

Overall, we proposed an albedo light-harvesting configuration for bifacial OSCs. AOTs based on periodic pyramids were fabricated using a silicon wet anisotropic etching method. By tuning the width of shadow mask, the spacing between pyramids was optimized, maximizing the front light blocking ability of AOT while barely affecting propagation of rear light. Although the integration of AOT endows transparent Ag film the asymmetrical optical transmission effect, considerable optical loss was found in ATEs.

Theoretical analysis and optical simulation reveal that the optical loss stems from the excitation and nonradiative damping of SPs. Therefore, ZnS film with high dielectric constant was used as an optical coupling layer to suppress the excitation of SPPs and increase the transmission of transparent Ag film. However, it is found that the generation of LSPRs at PDINO/Ag interface was simultaneously enhanced, which causes additional optical loss. Furthermore, the small bumps on Ag film feature much higher absorption coefficient compared to scattering coefficient. We thus doped organic emitter 2CzBN to PDINO film and revealed that the interaction between 2CzBN and SPs leads to SP-enhanced emission that recycles SP energy. With the regulation of SPs, the optimized ATE obtained front transmittance of 7% and rear transmission of 86%.

As a result of the asymmetrical optical transmission of ATE, bifacial OSCs exhibited impressive light-harvesting capability for both front and rear illumination. Along with the concentrated electric field distribution, the electromagnetic field induced by SPs was enhanced, which may contribute to more efficient energy transfer. Compared to a monofacial device with 18% efficiency, D18:Y6:Y6-1O–based bifacial OSCs obtained an outstanding PCE of 20.4% at an albedo factor of 0.2, which is ascribed to the synergistic effect of asymmetrical optical transmission and SP regulation. The outdoor testing and PDG analysis suggest that the optimized bifacial OSCs may perform much better in application scenarios with higher albedo factor. In addition, the feasibility of SP-ATE on flexible OSCs was also demonstrated.

In conclusion, we present a bifacial configuration that makes it possible for bifacial OSCs enabled performance to exceed that of monofacial counterparts. The findings herein may trigger a series of future work including morphology control of ultrathin Ag film, study on metal nanowire rear electrodes, and design of asymmetrical optical transmission structures, providing opportunities for breakthroughs in device performance of OSCs.

## MATERIALS AND METHODS

### Device fabrication

A silicon wafer with 200-nm SiO_2_ layer was cleaned by ultrasonication in deionized (DI) water, ethanol, and isopropanol for 15 min in sequence. The cleaned samples were dried by the nitrogen gun. The photoresist was spin-coated on the silicon wafer with the speed of 1000 rpm, yielding a uniform film with about 1-μm thickness. Then, the square patterns with the width of 40 μm and the spacing of 5 μm were formed by laser direct writing system (Microlab 4A-100-H) after the exposure and development. The prepared silicon wafer was etched by the dry etching process in a plasma reactor (Plasmalab 80 plus RIE, OXFORD Company) with 20 SCCM (standard cubic centimeter per minute) argon and 30 SCCM CF_3_, power of 150 W, and duration of 14 min. The criss-cross patterned mask was transferred to SiO_2_ layer after removing the residual photoresist by acetone. In the wet etching process, the alkaline solution was composed of KOH (20 g), ethanol (90 ml), and DI water (10 ml). The silicon wafer was reacted to inverted pyramid structures by the anisotropic etching at 65°C for about 8 hours in this etching solution. The utilization of ethanol can prevent the generated hydrogen gas from adhering the silicon wafer surface and affecting the uniformity of the etching process. The patterned silicon wafer can be obtained through removing the SiO_2_ hard mask by 10% hydrofluoric acid solution. The ITO glass substrate (Jinghui Tech., Wuhu) with a sheet resistance of 15 ohm sq^−1^ was cleaned by ultrasonication in Decon 90, DI water, ethanol, and isopropanol for 15 min in turn and dried at 110°C in an oven. The PEDOT:PSS was deposited on ITO layer at 4000 rpm for 40 s and annealed for 15 min at 140°C in a hot plate. The photovoltaic active layer solution was prepared by dissolving D18 (eFlexPV), Y6 (eFlexPV), and Y6-1O (eFlexPV) with a ratio of 1:1.12:0.48 in chloroform (Adams, 99.8%) and stirred at 60°C for 2 hours. The concentration is 5 mg ml^−1^ in terms of D18. The active layer was deposited on the PEDOT:PSS layer by spin coating active layer solution at 2500 rpm for 40 s and vapor-assisted annealed in a carbon disulfide atmosphere for 1 min in a nitrogen-filled glovebox. The PDINO solution (1-Materials) was dissolved in methanol with a concentration of 1 mg/ml and was spin-coated on the active layer at 4000 rpm for 30 s to serve as the electron transport layer. Eventually, a 0.5-nm Au film and a 12-nm Ag film were subsequently deposited on the aforementioned samples under a high vacuum thermal evaporation chamber with a base pressure of 2 × 10^−6^ torr. The effective area of the devices was determined to be 0.0725 cm^2^ by using the shadow mask. The ZnS optical coupling layer was also thermal-evaporated in vacuum. PC-based AOT was obtained through hot-press processing in Compact NanoImprint Tool (NIL Technology) for about 2 min. The pressure was set to 6 bar, and the temperature was set to 100°C. The pyramids could be transferred to PC film after cooling.

### Characterization

An ultraviolet–visible–near infrared spectrometer (PerkinElmer Lambda 950) was used to measure optical spectra. Surface morphologies of different silver films were characterized by SEM (ZEISS G500) and AFM (Veeco MultiMode V). The *J-V* characterization of different bifacial OSCs and monofacial devices was performed using Keithley 2400 source meter under simulated AM 1.5G solar irradiation at 100 mW cm^−2^ (2450_MPPT). EQE spectra were tested by a photomodulation spectroscopic setup (Enli Technology Co. Ltd., QE-R). Adhesion test was carried out by using peeling mode of universal testing machine (Instron, 34TM-50). The theoretical simulations about the thickness and admittance analysis of optical coupling layer were performed by using Essential Macleod.
