# Supplementary material for: 20.4% Power conversion efficiency from albedo-collecting organic solar cells under 0.2 albedo
Source: Sci Adv. 2024 Nov 1;10(44):eadp9439. doi: 10.1126/sciadv.adp9439 (PMC11529706; doi:10.1126/sciadv.adp9439)
Supplement: Supplementary file 1 — Figs. S1 to S17 Tables S1 to S5 [file sciadv.adp9439_sm.pdf]

Science Advances
AAAS

Supplementary Materials for

20.4% Power conversion efficiency from albedo-collecting organic solar cells under 0.2 albedo

Hao Ren *et al.*

Corresponding author: Jian-Xin Tang, jxtang@must.edu.mo; Jing-De Chen, jdchen@suda.edu.cn; Yan-Qing Li, yqli@phy.ecnu.edu.cn

Sci. Adv. **10**, eadp9439 (2024)
DOI: 10.1126/sciadv.adp9439

This PDF file includes:

Figs. S1 to S17
Tables S1 to S5

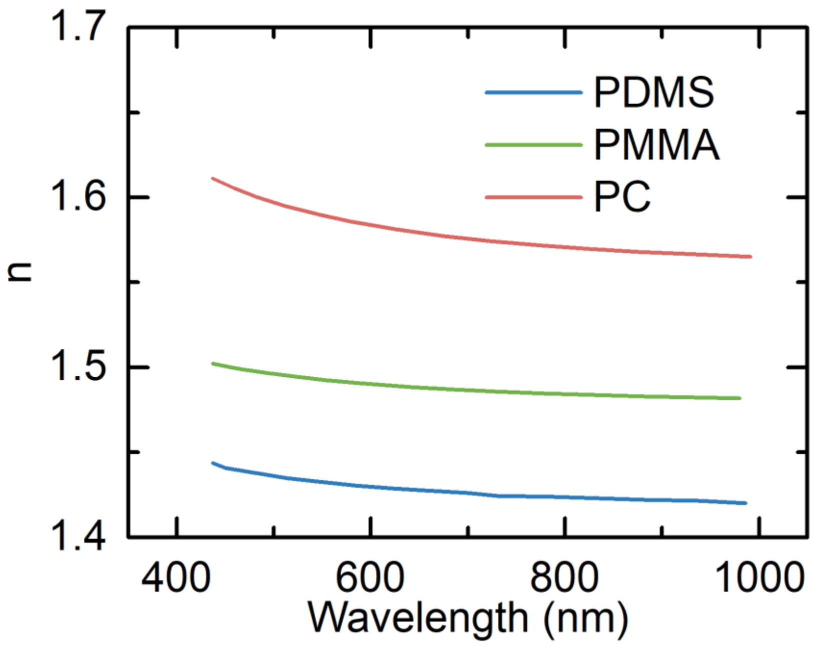

Fig. S1. Impact of materials selection on refractive index. Refractive index of PDMS, PMMA, and PC.

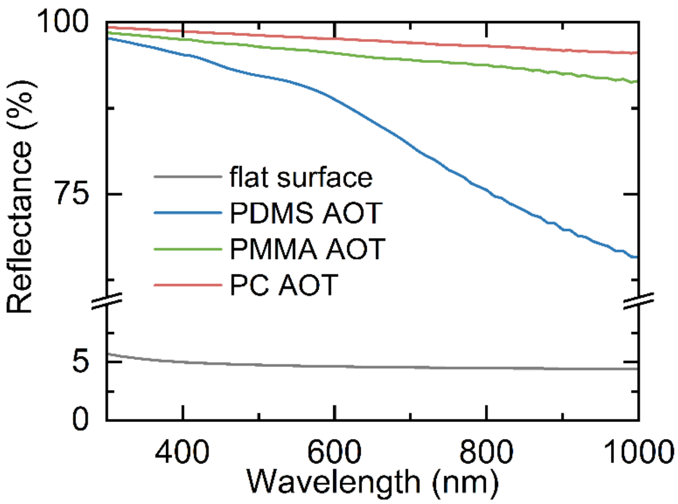

Fig. S2. Impact of transparent material on reflectance. Simulated reflection spectra of flat surface and AOTs based on various transparent materials.

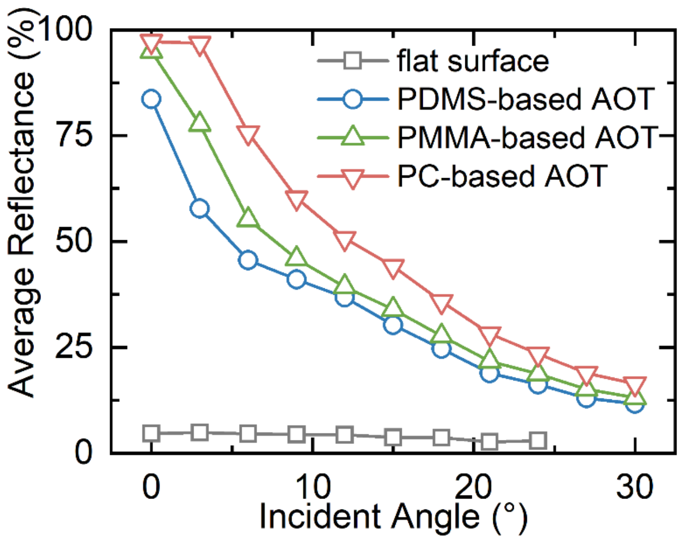

Fig. S3. Impact of transparent material on reflectance. Simulated average reflectance of flat surface and AOTs versus the angle of incidence.

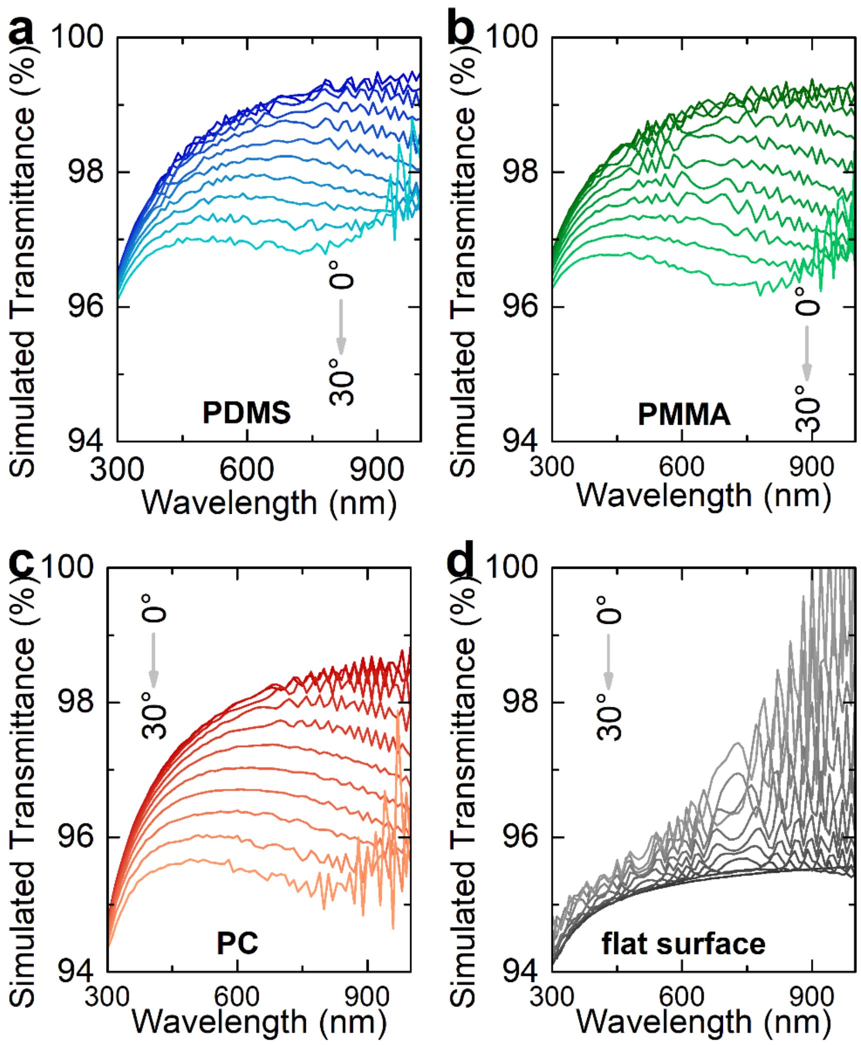

Fig. S4. Impact of transparent material on transmittance. Simulated transmission spectra of (a) PDMS- based AOT, (b) PMMA- based AOT, (c) PC-based AOT, and (d) flat surface at different incident angles and wavelength under rear illumination.

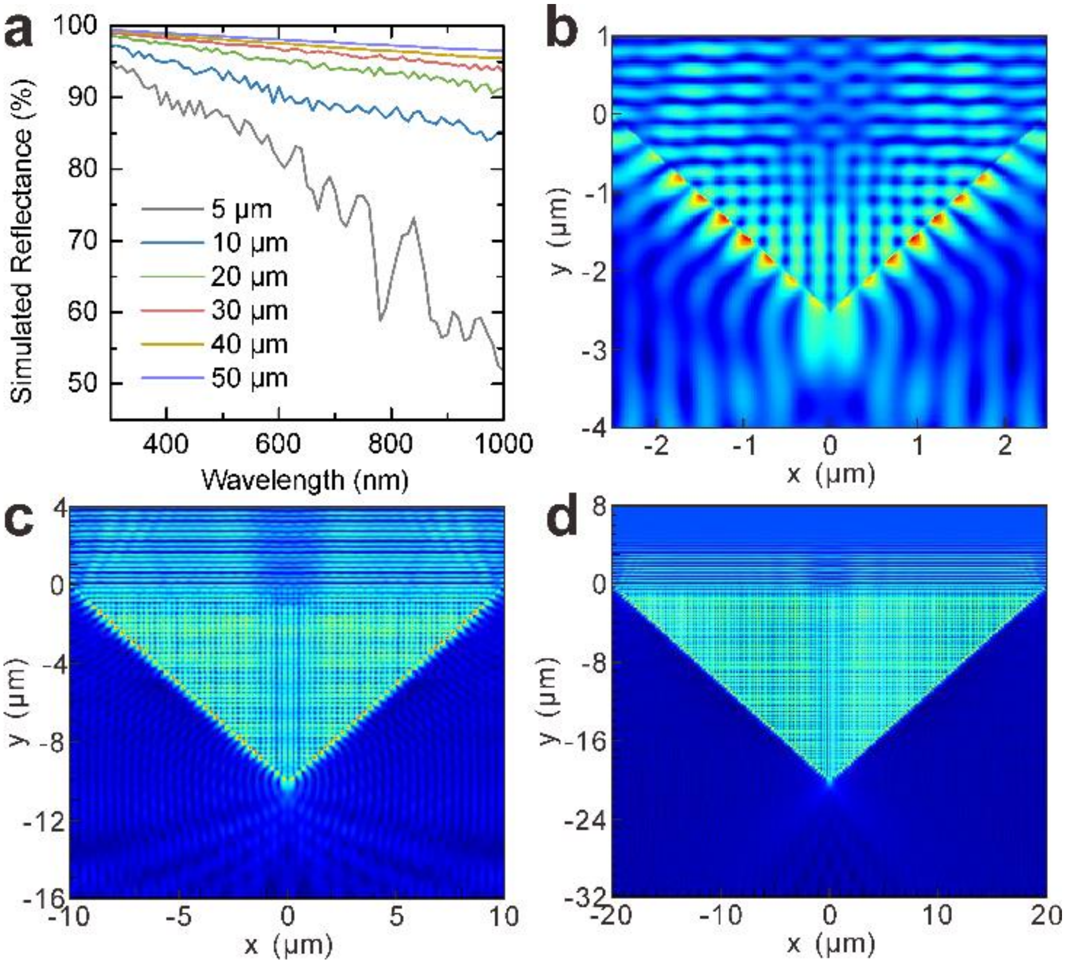

Fig. S5. Impact of pyramid on optical property of PC-based AOT. (a) Simulated reflection spectra PC-based AOT with different periods. Simulated electric field distribution profiles of PC-based AOT based on pyramid array with period of (b) 5 µm, (c) 20 µm, and (d) 40 µm.

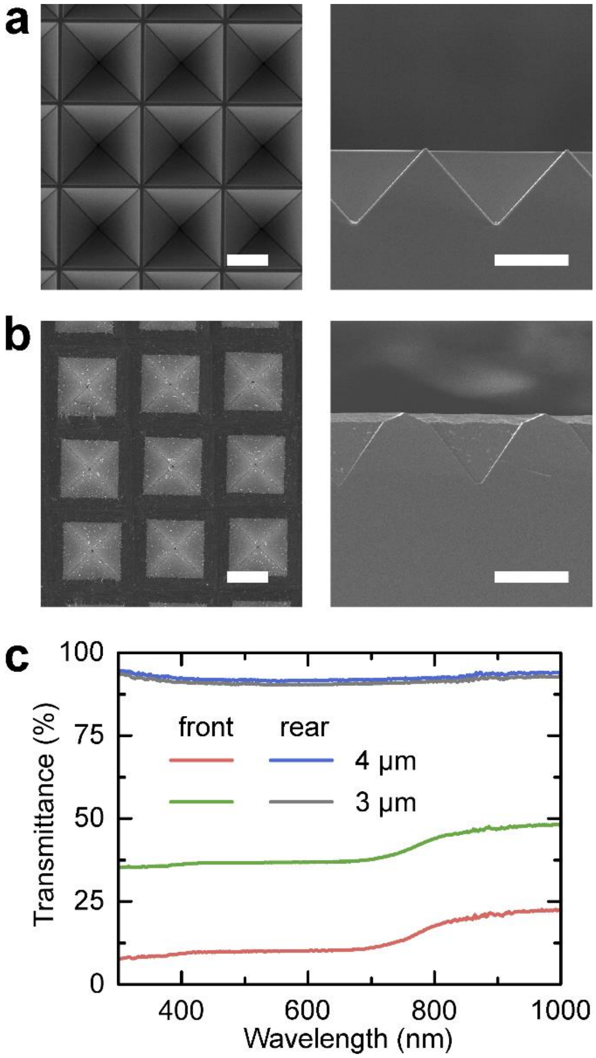

Fig. S6. Measurements of silicon mold with different width. Top-view and cross-sectional SEM images of silicon mold processed using shadow masks with width of (a) 4 μm and (b) 3 μm.（c) Transmission spectra of AOT processed with reduced width of shadow mask.

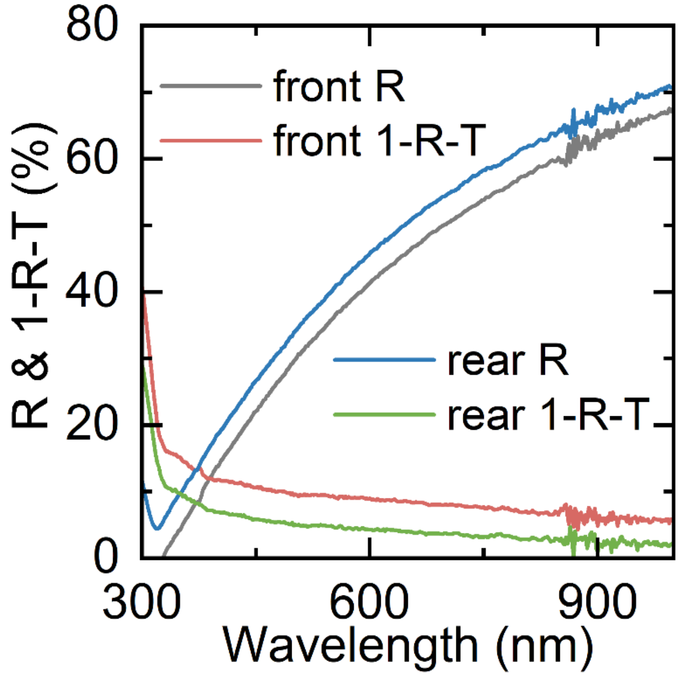

Fig. S7. Impact of illumination direction on 1-R-T. Reflection spectra and 1-R-T spectra of bare Ag with different illumination direction.

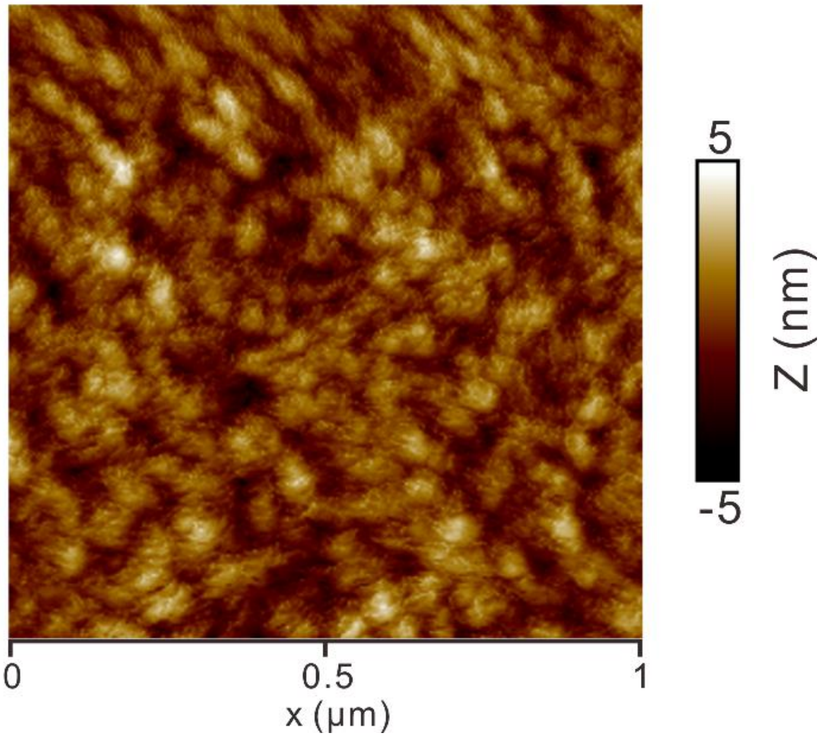

Fig. S8. Surface morphology of active layer. AFM height image of D18:Y6:Y6-1O**.**

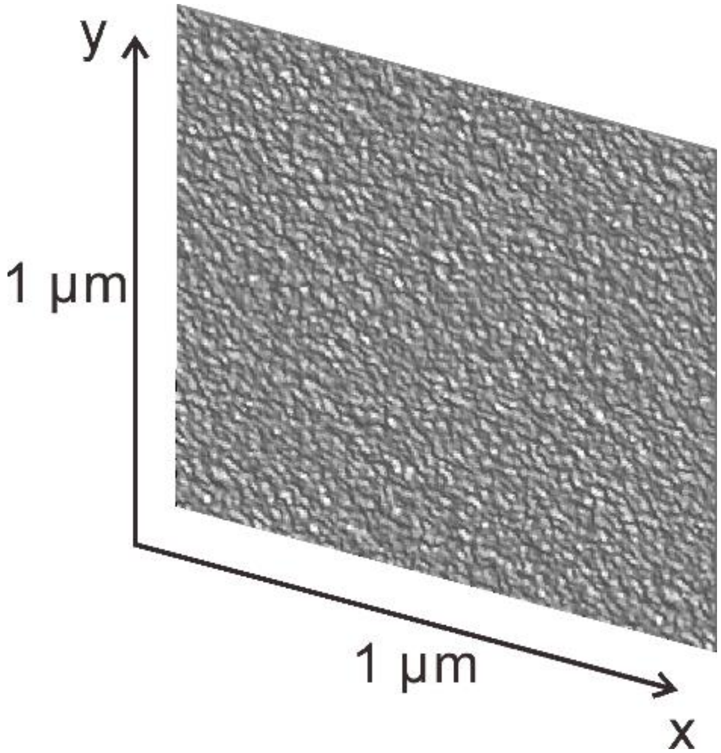

Fig. S9. Modeling of rough Ag film. The graph of the rough surface use in optical simulation.

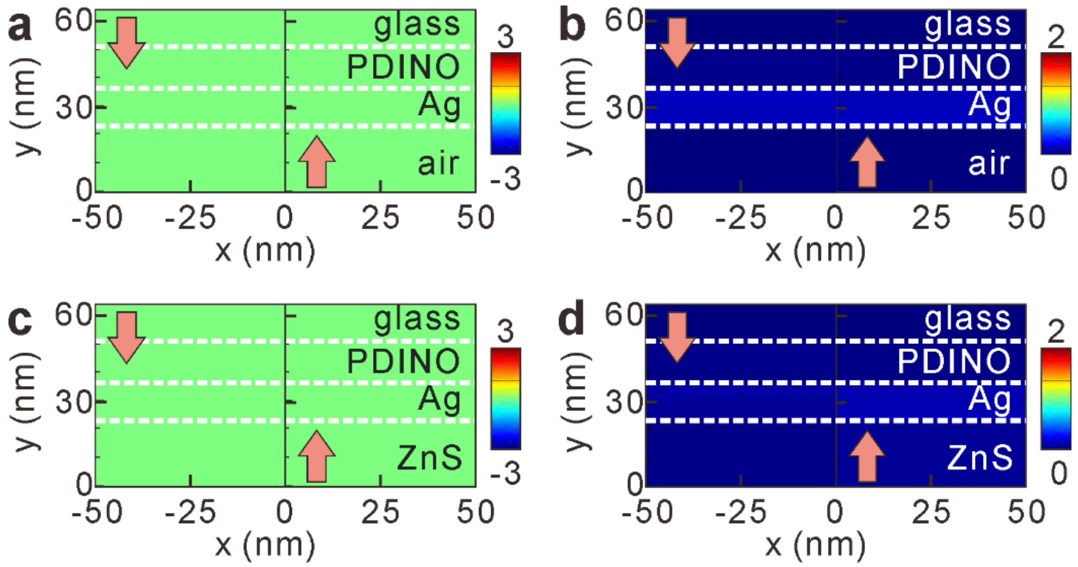

Fig. S10. Impact of optical coupling layer on E_y and absorption distribution. Simulated (a) E_y and (b) absorption distribution profiles of flat Ag film. Simulated (c) E_y and (d) absorption distribution profiles of flat Ag film with optical coupling layer.

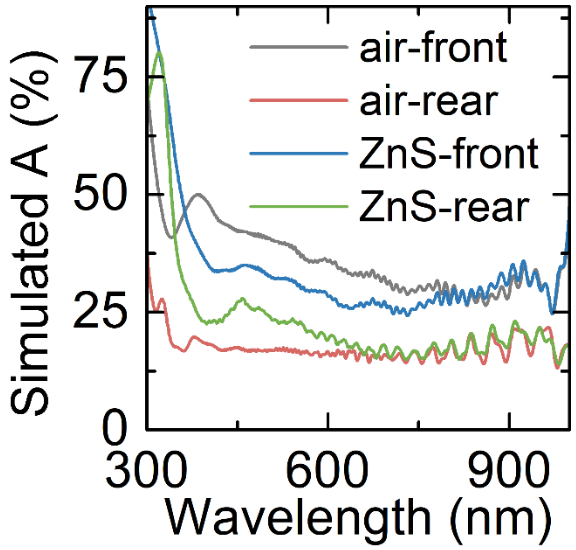

Fig. S11. Impact of optical coupling layer on absorption. Simulated absorption spectra of ATEs integrated with air and ZnS.

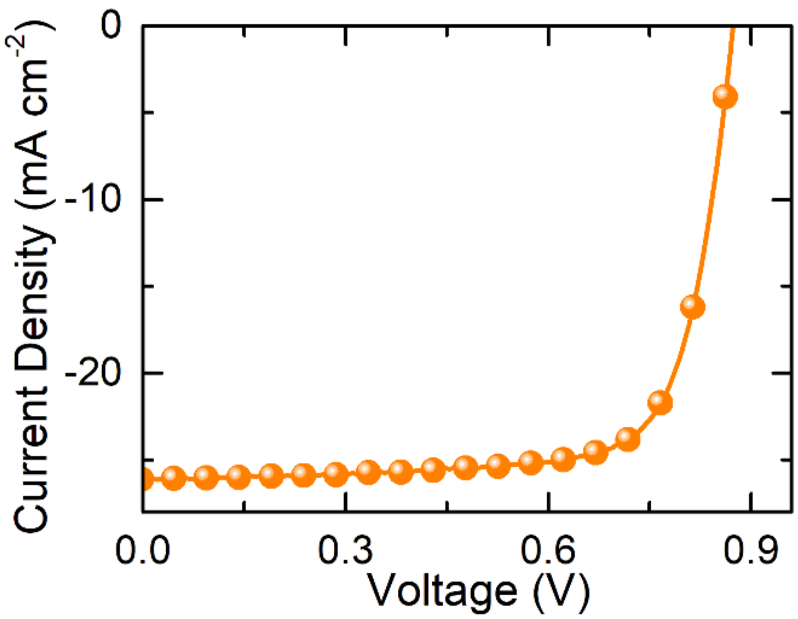

Fig. S12. Photovoltaic performance of opaque OSC. *J-V* curve of opaque OSC under the AM 1.5G illumination with power intensity of 100 mW cm^{-2}.

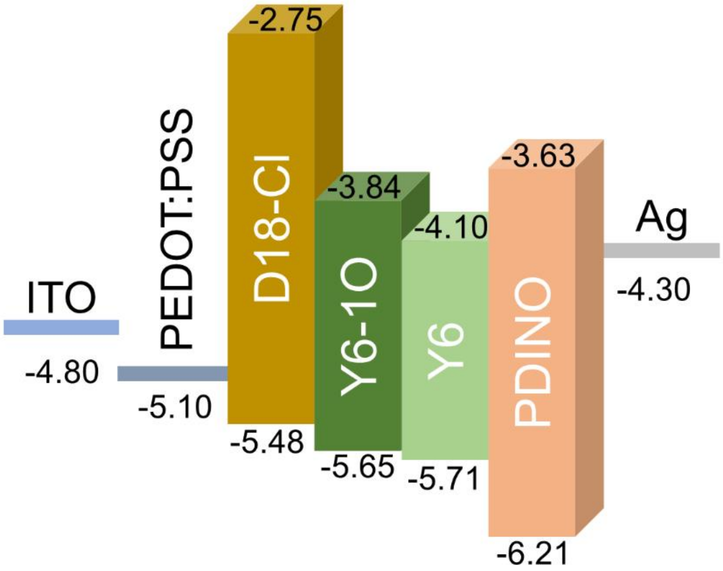

Fig. S13. Energy level structure of OSC. Energy level diagram of the whole device.

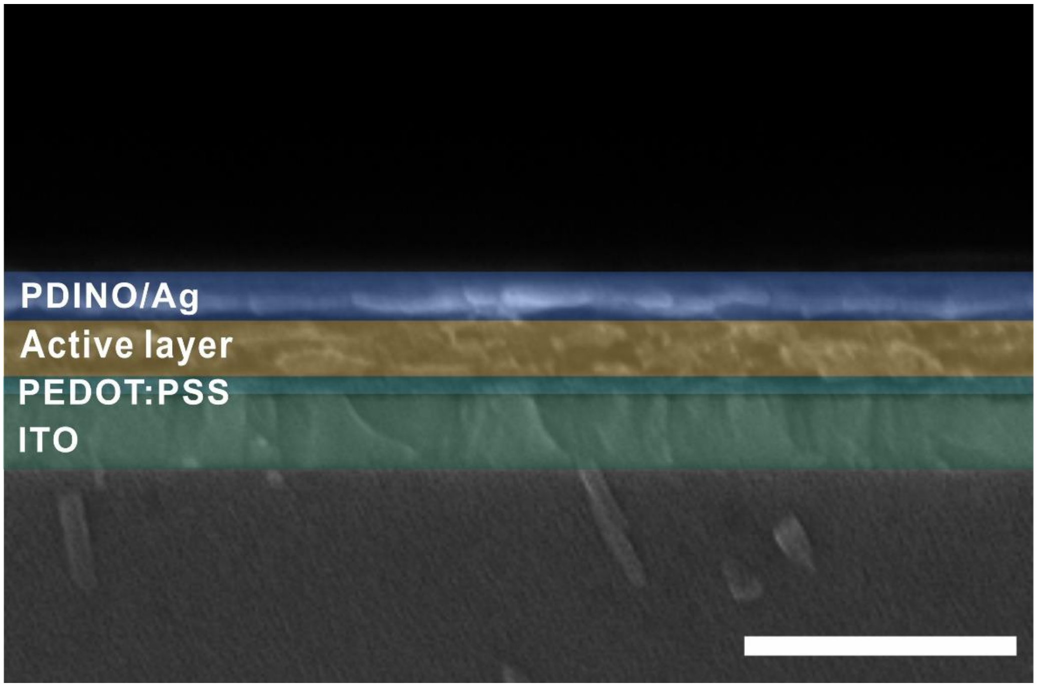

Fig. S14. Measurements of active layer thickness. Cross-section SEM images of bifacial OSCs under optimized conditions. The scale bar is 500 nm.

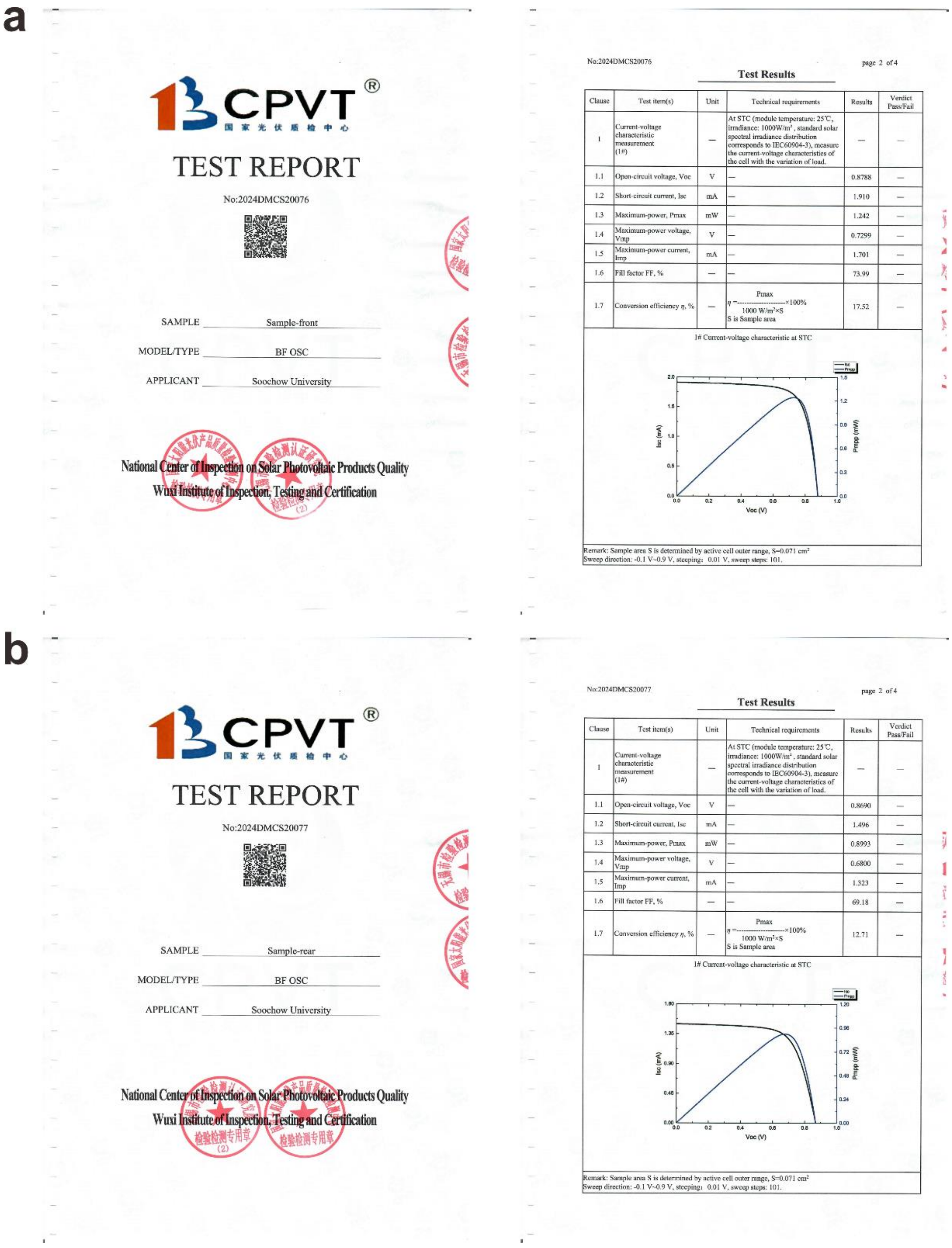

a

CPVT®

国家光伏质检中心

TEST REPORT

No:2024DMCS20076

SAMPLE Sample-front

MODEL/TYPE BF OSC

APPLICANT Soochow University

National Center of Inspection on Solar Photovoltaic Products Quality

Wuxi Institute of Inspection, Testing and Certification

No:2024DMCS20076 page 2 of 4

Test Results

Clause	Test item(s)	Unit	Technical requirements	Results	Verdict Pass/Fail
1	Current-voltage characteristic measurement (1#)	—	At STC (module temperature: 25℃, irradiance: 1000W/m², standard solar spectral irradiance distribution corresponds to IEC60904-3), measure the current-voltage characteristics of the cell with the variation of load.	—	—
1.1	Open-circuit voltage, Voc	V	—	0.8788	—
1.2	Short-circuit current, Isc	mA	—	1.910	—
1.3	Maximum-power, Pmax	mW	—	1.242	—
1.4	Maximum-power voltage, Vmp	V	—	0.7299	—
1.5	Maximum-power current, Imp	mA	—	1.701	—
1.6	Fill factor FF, %	—	—	73.99	—
1.7	Conversion efficiency η, %	—	$\eta = \frac{Pmax}{1000\ W/m^2 \times S} \times 100\%$ S is Sample area	17.52	—

1# Current-voltage characteristic at STC

Remark: Sample area S is determined by active cell outer range, S=0.071 cm²

Sweep direction: -0.1 V~0.9 V, steeping: 0.01 V, sweep steps: 101.

b

CPVT®

国家光伏质检中心

TEST REPORT

No:2024DMCS20077

SAMPLE Sample-rear

MODEL/TYPE BF OSC

APPLICANT Soochow University

National Center of Inspection on Solar Photovoltaic Products Quality

Wuxi Institute of Inspection, Testing and Certification

No:2024DMCS20077 page 2 of 4

Test Results

Clause	Test item(s)	Unit	Technical requirements	Results	Verdict Pass/Fail
1	Current-voltage characteristic measurement (1#)	—	At STC (module temperature: 25℃, irradiance: 1000W/m², standard solar spectral irradiance distribution corresponds to IEC60904-3), measure the current-voltage characteristics of the cell with the variation of load.	—	—
1.1	Open-circuit voltage, Voc	V	—	0.8690	—
1.2	Short-circuit current, Isc	mA	—	1.496	—
1.3	Maximum-power, Pmax	mW	—	0.8993	—
1.4	Maximum-power voltage, Vmp	V	—	0.6800	—
1.5	Maximum-power current, Imp	mA	—	1.323	—
1.6	Fill factor FF, %	—	—	69.18	—
1.7	Conversion efficiency η, %	—	$\eta = \frac{Pmax}{1000\ W/m^2 \times S} \times 100\%$ S is Sample area	12.71	—

1# Current-voltage characteristic at STC

Remark: Sample area S is determined by active cell outer range, S=0.071 cm²

Sweep direction: -0.1 V~0.9 V, steeping: 0.01 V, sweep steps: 101.

Fig. S15. Copy of bifacial OSCs certificate results by the National Center of Supervision & Inspection on Solar Photovoltaic Products Quality of China (CPVT). Certified reports of the champion bifacial OSCs under the (a) front and (b) rear illumination, respectively.

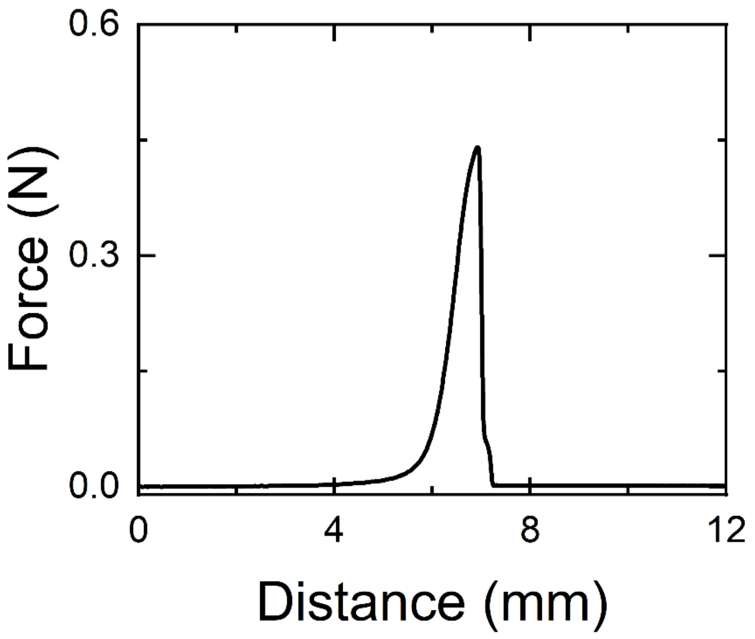

Fig. S16. Physical contact of AOT. Adhesion measurement between patterned PC and Ag electrode.

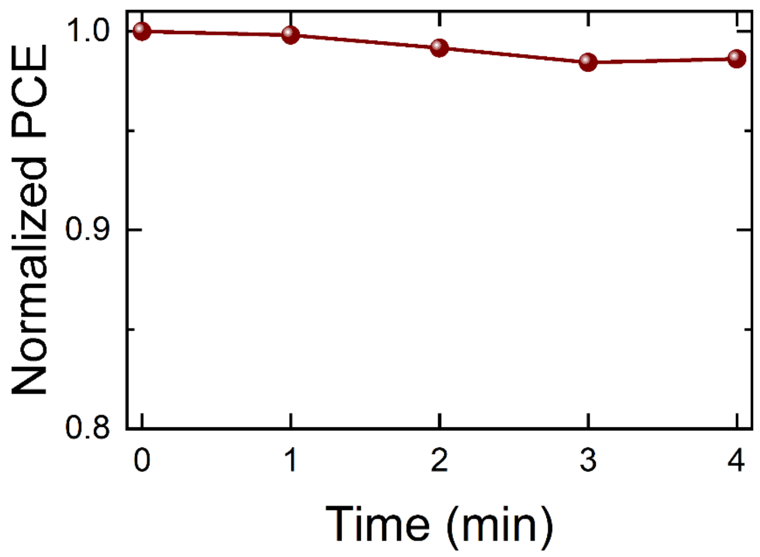

Fig. S17. Thermal stability of bifacial OSCs. Normalized PCE of bifacial OSCs under continuous heating at 100 °C.

Table S1. Photovoltaic performance of bifacial OSCs. Photovoltaic parameters for various bifacial OSCs under 100 mW cm^{-2} AM 1.5G illumination. The average PCE (PCE_{ave}) were obtained from 16 devices.

Rear electrode	Illumination direction	V_{OC} (V)	J_{SC} (mA cm^{-2})	FF (%)	PCE (%)	PCE_{ave} (%)
12 nm Ag	Front	0.87	23.12	74.59	15.08	14.82
	Rear	0.85	14.73	72.61	9.08	8.76
ATE	Front	0.87	25.21	74.79	16.47	16.03
	Rear	0.84	17.09	72.29	10.42	9.7
SP-ATE	Front	0.87	27.12	74.29	17.57	17.25
	Rear	0.84	21.13	72.24	12.75	12.38

Table S2. Optical characteristics of bifacial OSCs. Optical parameters for various bifacial OSCs under 100 mW cm^{-2} AM 1.5G illumination.

Rear electrode	Illumination direction	AVT (%)	PCE (%)	LUE (%)
12 nm Ag	Front	13.58	15.08	2.05
	Rear	13.78	9.08	1.25
ATE	Front	3.70	16.47	0.61
	Rear	15.43	10.42	1.60
SP-ATE	Front	4.56	17.57	0.80
	Rear	19.43	12.75	2.48

Table S3. Photovoltaic performance of bifacial OSCs. Photovoltaic parameters of D18:Y6(Y6-1O)-based bifacial OSCs with different D/A ratios. The average PCE (PCE_{ave}) were obtained from 16 devices.

D/A ratio	V_{OC} (V)	J_{SC} (mA cm^{-2})	FF (%)	PCE (%)	PCE_{ave} (%)
1:1.8	0.87	29.35	75.08	19.26	18.78
1:1.6	0.88	30.85	74.83	20.20	19.82
1:1.4	0.88	29.85	74.13	19.39	19.01
1:1.2	0.88	28.76	72.95	18.38	17.89

Table S4. Photovoltaic performance of bifacial OSCs. Photovoltaic parameters of D18:Y6(Y6-1O)-based bifacial OSCs with different active layer thicknesses. The average PCE (PCE_{ave}) were obtained from 16 devices.

Thickness (nm)	V_{OC} (V)	J_{SC} (mA cm^{-2})	FF (%)	PCE (%)	PCE_{ave} (%)
145	0.87	29.20	71.75	18.21	17.88
121	0.87	29.05	73.13	18.54	18.16
103	0.88	30.97	74.75	20.35	19.97
80	0.88	29.33	75.79	19.60	19.19
58	0.88	27.12	75.16	17.92	17.63

Table S5. Photovoltaic performance of bifacial OSCs. Photovoltaic parameters of D18:Y6(Y6-1O)-based bifacial OSCs with different additives. The average PCE (PCE_{ave}) were obtained from 16 devices.

Additive	V_{OC} (V)	J_{SC} (mA cm^{-2})	FF (%)	PCE (%)	PCE_{ave} (%)
FN	0.87	30.08	74.98	19.57	18.98
CN	0.88	28.15	79.55	19.70	19.16
DIO	0.85	28.77	77.77	18.95	18.00
